# Tailoring Sieving Pores and Electrochemical Interface Intercalation for Mechanically Resilient Recycled Micro‐Silicon Anodes

**DOI:** 10.1002/advs.202517656

**Published:** 2025-12-05

**Authors:** Yunan Wei, Ruilin Wu, Shixin Liu, Han Liang, Runwei Mo

**Affiliations:** ^1^ School of Mechanical and Power Engineering East China University of Science and Technology Shanghai 200037 China; ^2^ Shanghai Key Laboratory of Intelligent Sensing and Detection Technology East China University of Science and Technology Shanghai 200237 China

**Keywords:** electrochemical intercalation, lithium‐ion battery, non‐destructive testing‌, silicon, structural design

## Abstract

To meet the demands of high‐energy lithium‐ion batteries, micron‐sized silicon (Si) anodes need to have high capacity, low expansion, long life, and fast charging characteristics. However, their industrialization is constrained by the fundamental contradiction between particle deformation and charge transfer. Here, an innovative strategy is proposed, using micron‐sized Si recovered from photovoltaic waste as raw material, combined with electrochemical lithium alloying and rapid heating, reacting with CO_2_ and acid washing to obtain a sieve‐like porous structure design to overcome mechanical dynamic limitations. The structural design achieves extremely high capacity and low electrode expansion rate (10.8% at 2493 mAh g^−1^ and 8.72 mAh cm^−2^), excellent rate performance (1257 mAh g^−1^ and 4.4 mAh cm^−2^ at 2 A g^−1^ over 1000 cycles), and ultra‐low capacity decay rate (0.016% per cycle). The pouch cell achieves a capacity retention rate of 91.5% over 500 cycles and a record‐breaking volumetric energy density of 1428 Wh L^−1^. In addition, a non‐destructive testing method based on the dp/|dQ| peak as a fault warning signal is also developed, which establishes a new method for early safety warning for Si‐based batteries. This work provides an efficient and environmentally friendly solution for the resource utilization of photovoltaic silicon waste.

## Graphical abstract

Here, an innovative strategy is proposed, using micron‐sized Si recovered from photovoltaic waste as raw material, combined with electrochemical lithium alloying, reacting with CO_2_ to obtain sieve‐like porous structure design to overcome mechanical dynamic limitations. The pouch cell achieves a capacity retention rate of 91.5% over 500 cycles and a record‐breaking volumetric energy density of 1428 Wh L^−1^.

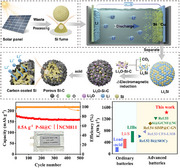

## 1 Introduction

Against the backdrop of global carbon neutrality, the rapid development of solar power generation has led to a large amount of high‐purity photovoltaic silicon waste, resulting in resource waste and environmental pollution.^[^ ^1^, ^2^, ^3^ ^]^ To meet the demand for high‐energy‐density energy storage in fields such as electric vehicles, Micron‐sized silicon (Si) anodes with a theoretical capacity ten times that of graphite have become a hot topic in the research of high‐energy‐density lithium‐ion batteries.^[^ ^4^, ^5^ ^]^ Developing high‐performance micron‐sized silicon‐based anodes using photovoltaic Si waste as raw material, which is abundant, low‐cost, and of good purity, can not only significantly reduce the cost of lithium‐ion batteries and increase energy density to support the transition to clean energy, but also achieve green, high‐value recycling of waste from the photovoltaic industry chain. This has dual strategic significance for promoting the achievement of the “dual carbon” goals in terms of both economic and environmental protection.

Micron‐sized Si has emerged as a key anode for high‐energy‐density lithium‐ion batteries owing to its extremely high theoretical specific capacity (4200 mAh g^−1^) and low lithium lithiation potential (0.4 V vs Li/Li). However, it still faces two fundamental challenges: i) Si exhibits significant volume expansion (≈300%), which not only causes active material cracking and electrical contact failure, but also leads to repeated rupture and regeneration of the solid electrolyte interphase (SEI) film, severely impairing cycle life and coulombic efficiency; ii) as a semiconductor material, Si has low intrinsic conductivity, limiting charge transport and rate performance. To overcome the aforementioned obstacles, combining silicon with carbon materials that exhibit excellent conductivity and stability to design silicon–carbon composite materials and porous structures has become the most promising research direction at present. These materials aim to utilize the carbon components to achieve three functions: i) constructing an efficient conductive network to enhance overall electrical conductivity; ii) providing a buffer space or framework to alleviate silicon's volume expansion stress; iii) stabilizing the formation of the SEI layer during charging and discharging. It is worth noting that the selection of carbon sources and structural design are critical to material performance, cost, and environmental footprint.

Although the silicon–carbon composite strategy offers an important solution, it still faces a core contradiction in achieving the goal of micron‐sized Si‐based anode materials that simultaneously possess high capacity, low volume expansion, long cycle life, and excellent fast‐charging capabilities. On the one hand, designs aimed at enhancing mechanical stability and suppressing expansion damage (such as dense carbon coating and large cavities) often significantly hinder lithium‐ion diffusion, sacrificing rate performance and fast charging capabilities. On the other hand, designs pursuing high dynamic performance (such as high porosity open structures and thin‐layer carbon networks) are prone to structural weakness or excessive Si exposure, leading to continuous SEI growth and structural collapse, thereby shortening cycle life.

Additionally, the complex and intricate structures required for high‐performance Si‐based materials (such as nanostructure, porosity, and special coatings) often rely on high‐cost or environmentally unfriendly preparation processes, severely limiting their feasibility for large‐scale industrial production. Although Si anodes have enormous potential for high capacity, their practical application and widespread industrialization are severely constrained by the inherent contradiction between particle deformation and efficient charge transfer. Current technological approaches are generally caught in the fundamental dilemma of being unable to simultaneously achieve both mechanical stability and electrochemical kinetic performance. Therefore, it remains a fundamental challenge to break through the trade‐off between mechanical stability and kinetic performance, and to develop high‐performance micron‐sized Si‐based anodes that can be produced on a large scale.

**Figure 1:**
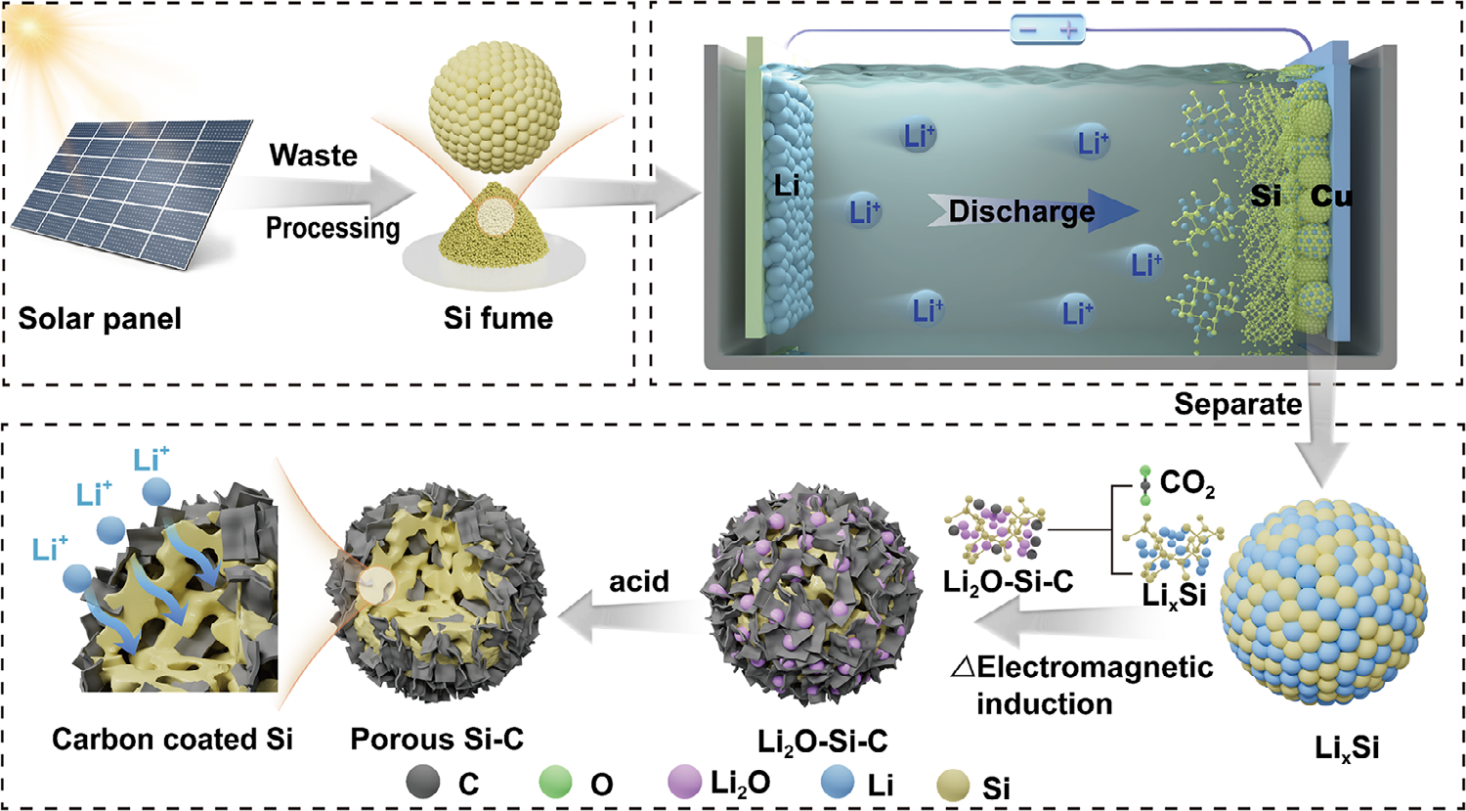
Process flow chart for synthesizing sieve‐like porous silicon/carbon structures through electrochemical alloying, CO_2_ rapid induction heating reaction and PVDF carbonization.

Herein, we propose an innovative strategy, using micron‐sized Si recovered from photovoltaic waste as raw material, combined with electrochemical lithium alloying and rapid heating, reacting with CO_2_ and acid washing to obtain a sieve‐like porous structure design to overcome mechanical dynamic limitations (**Figure** **1**). This design mainly uses CO_2_ as the carbon source, and PVDF carbonization provides a small amount of carbon. This unique sieve‐like porous silicon–carbon composite material (P‐Si@C) structure not only features an inner sieve‐pore structure that can accommodate the deformation of silicon, allowing ions to rapidly transport through reserved gaps within the pores, but also induces stress‐voltage coupling effects by mechanically confining silicon within the nanopores, thereby achieving stable and rapid (de‐alloying) reactions. P‐Si@C achieves extremely high capacity and very low electrode expansion rate (10.8% at 2493 mAh g^−1^ and 8.72 mAh cm^−2^), excellent rate performance (1257 mAh g^−1^ and 4.4 mAh cm^−2^ at 2 A g^−1^ over 1000 cycles), and ultra‐low capacity decay rate (0.016% per cycle). This pouch full cell shows an ultra‐high energy density of 1428 Wh L^−1^, which exceeds previously reported silicon‐based batteries. In addition, we also have innovatively developed a non‐destructive testing method based on the dp/|dQ| peak as a fault warning signal, which establishes a new method for early safety warning for Si‐based batteries. This work provides an efficient and clean new method for constructing high‐performance Si‐based structures using photovoltaic Si waste and carbon dioxide resources, which also opens up new directions for establishing a Si‐based pressure monitoring and early warning analysis system.

## 2 Results and Discussion

During the electrochemical lithiation process, as Li migrates, the Si crystals within the Si particles gradually transform into Li_x_Si alloys. The formula for the electrochemical lithiation reaction is as shown in Equation (1). Electrochemical lithiation enables lithium ions to enter micron‐sized Si particles in an orderly manner, laying a solid foundation for the subsequent rapid heating reaction with CO_2_ and the sieve‐like porous structure formed by acid washing. To fully complete the electrochemical lithiation process, the discharge voltage inside the electrolytic cell is set to 0.01 V (with lithium foil as the counter electrode). The obtained Li_x_Si alloy was transferred to an electromagnetic induction furnace protected by argon gas. Li_x_Si reacted rapidly with CO_2_ to obtain an intermediate containing the by‐product (Li_2_O), and the reaction equation was shown in Equation (2).^[^ ^6^ ^]^ Among them, CO_2_ provides the main carbon source, and the carbonization of PVDF also generates a small amount of carbon. This makes the carbon distribution in the material uniform and abundant. After acid washing to remove oxides and impurities, a sieve‐shaped porous silicon‐carbon composite (P‐Si@C) material is made.

(1) $$\mathrm{Si}+\mathrm{xL}i^{-}+xe^{-}\to Li_{x}\mathrm{Si}\left(0\leq x\leq 4.4\right)$$

(2) $$2Li_{x}\mathrm{Si}+\frac{x}{2}CO_{2}\to \mathrm{xL}i_{2}O+\frac{x}{2}C+2\mathrm{Si}\left(0\leq x\leq 4.4\right)$$

**Figure 2:**
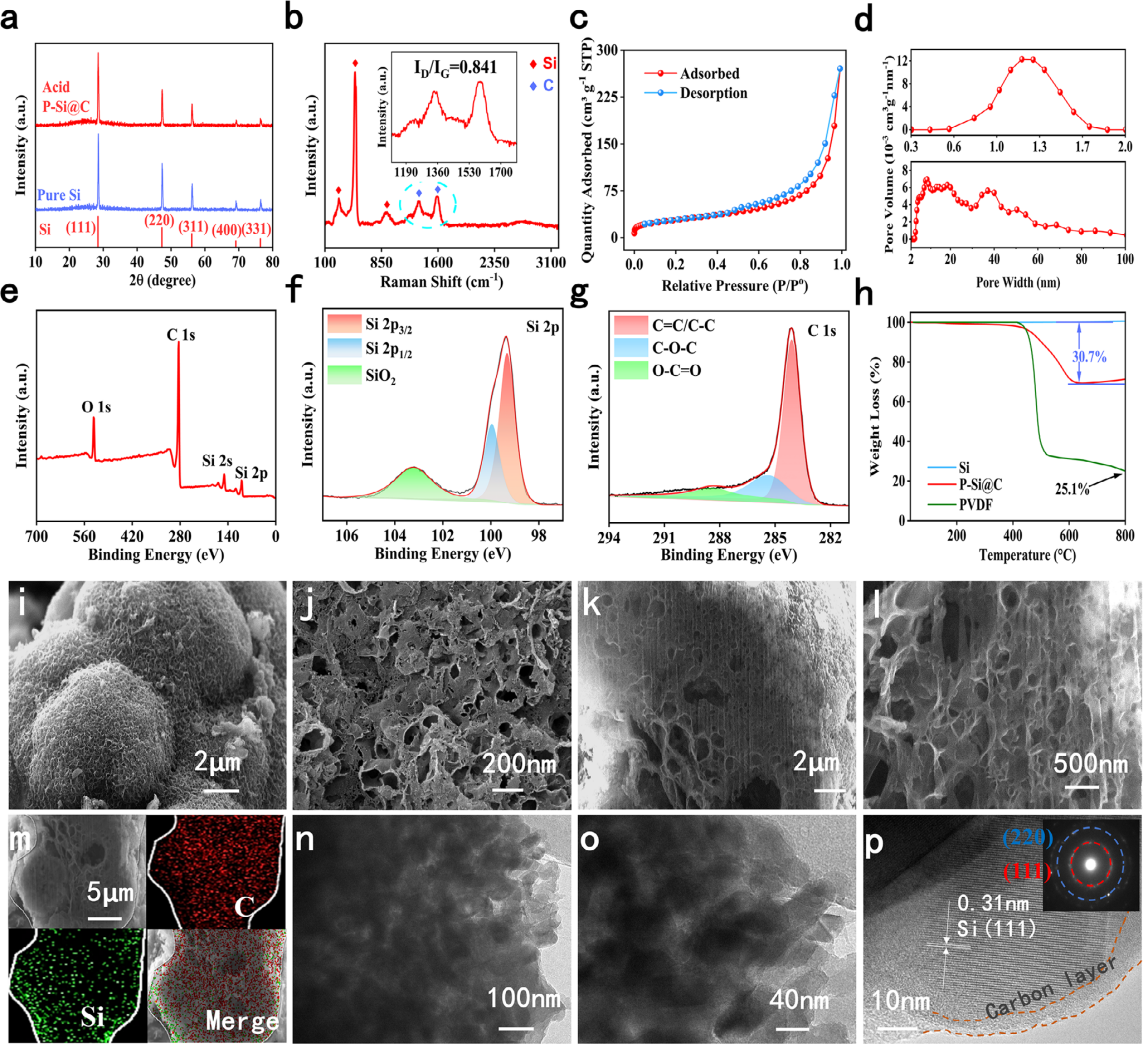
Phase characterization and morphological properties of P‐Si@C. a) XRD patterns of Si and P‐Si@C after pickling. b) Raman spectra of P‐Si@C. c,d) BET absorption‐release curve and the pore size distribution of P‐Si@C. e) XPS spectra of P‐Si@C, f) C 1s, g) Si 2p. h) TGA diagrams of P‐Si@C, PVDF and Si. i,j) SEM images of P‐Si@C. k–m) SEM and EDS images of the cross section of P‐Si@C obtained by FIB processing. n,o) TEM image of P‐Si@C, (o) is the high magnification local magnification image of (n). p) HRTEM of P‐Si@C.

Figure S1 (Supporting Information) shows the XRD pattern of the intermediate product without acid washing, indicating the presence of LiF, Si, Li_2_SiO_3_, and some less prominent trace impurity peaks.^[^ ^7^, ^8^, ^9^ ^]^ Among them, LiF is a component of SEI formed during the electrochemical lithiation process, Si is a product caused by Li_x_Si rapid heating reaction with CO_2_, and Li_2_SiO_3_ is a by‐product of this reaction. **Figure** **2**a shows the XRD patterns of P‐Si@C and pure Si after acid washing. The three main peaks are at 28.5°, 47.4°, and 56.3°, corresponding to the (111), (220), and (311) crystal planes of crystalline Si,^[^ ^10^, ^11^ ^]^ indicating that the reaction has been completely carried out and that oxides and impurities have been removed by the acid. The Raman spectrum of P‐Si@C is shown in Figure 2b, where the characteristic peaks of Si appear at 284, 499, and 916 cm^−1^, demonstrating the presence of crystalline Si in composite.^[^ ^12^, ^13^ ^]^ Additionally, it can be observed that there are D peaks caused by carbon layer defects and hybridization, as well as G peaks caused by C─C bonds.^[^ ^14^ ^]^ The intensity ratio of the D peak to the G peak can reflect the degree of graphitization of carbon layer. From the local enlargement diagram, it was found that the D peak and G peak are located at 1356 and 1587 cm^−1^, respectively, and the ratio of I_D_/I_G_ is ≈0.841, indicating that the carbon layer of the material has a high degree of graphitization, which was conducive to enhancing the conductivity of composite.^[^ ^15^ ^]^ As can be seen in Figure 2c,d, the specific surface area estimated based on isotherms is 101.7 m^2^ g^−1^, with pore sizes distributed in the range of 0.3–100 nm, indicating that the P‐Si@C has a rich sieving pore and nanopore structure to synergistically regulate electrolyte permeation and lithium‐ion transport behavior. In common carbonate‐based electrolytes, the diameter of solvated lithium ions typically ranges ≈1.5 nm. The 0.3–1.5 nm sieve pores effectively restrict access by solvated lithium ions (diameter ≈1.5 nm), promoting SEI film formation primarily on the electrode surface and enhancing interfacial stability. Additionally, the 1.5–100 nm nanopores provide rapid migration pathways for lithium ions, mitigating the hindrance to ion transport imposed by the sieve pore region. This approach significantly improves rate performance while ensuring electrode structural stability. Considering the influence of conductive carbon black in the sample, the mixture was prepared at a ratio of Si: PVDF: conductive carbon black = 7:1.5:1.5, followed by carbonization under identical heating conditions. It can be observed that the pore properties of silicon–carbon materials without electrochemical lithiation and CO_2_ reaction (w‐Si@C) exhibit significant differences compared to P‐Si@C (Figure S2, Supporting Information). BET results indicate that w‐Si@C exhibits virtually no discernible pores with a specific surface area of only 40.4 m^2^ g^−1^, whereas P‐Si@C possesses a rich pore structure with a specific surface area as high as 101.7 m^2^ g^−1^. This sieve‐like porous structure exhibits size effects, enabling thorough electrolyte wetting and allowing lithium ions to freely traverse the pore. Additionally, these nano‐pores serve as rapid transport channels for lithium ions, promoting their movement and dispersion, and mitigating damage caused by Si expansion, which effectively enhances the rate performance and cycling stability.^[^ ^16^ ^]^

To characterize the elemental composition and chemical state of P‐Si@C, we employed X‐ray photoelectron spectroscopy (XPS) technology. As shown in Figure 2e, the spectrum exhibits four distinct characteristic peaks: O 1s, C 1s, Si 2s, and Si 2p, indicating the presence of Si, C, and O elements in the sample, with no other impurity elements detected.^[^ ^17^, ^18^ ^]^ As shown in Figure 2f, the Si 2p spectrum peak can be divided into three peaks: Si 2p_3/2_, Si 2p_1/2_, and SiO_2_, corresponding to peak values at 99.32, 99.96, and 103.21 eV, respectively.^[^ ^19^ ^]^ It is worth noting that SiO_2_ may originate from residues from the acid washing process or air interference during the detection process, but this peak is not significant. The C 1s spectrum can be divided into C═C/C─C (284.10 eV), C─O─C (285.38 eV), and O─C═O (288.42 eV), with the C═C/C─C peak being the main peak (Figure 2g).^[^ ^20^ ^]^ Considering the influence of conductive carbon black and PVDF, we performed XPS analysis on w‐Si@C for comparison (Figure S3, Supporting Information). From the XPS results, there is no significant difference in the elemental composition. There are only some differences in SiO_2_ caused by acid washing, indicating that the carbon produced by the reaction of Li_x_Si with CO_2_ is essentially similar to that produced by carbonization of conductive carbon black and PVDF. In addition, we conducted thermogravimetric analysis (TGA) tests to determine the Si and carbon (C) content in the samples. The test conditions were conducted in an air atmosphere, with the temperature rising from 25 to 800 °C. As shown in Figure 2h, the curve for pure Si is relatively flat and exhibits a slight increase after 650 °C, which is primarily attributed to the oxidation process of Si.^[^ ^21^ ^]^ The curve for P‐Si@C begins to drop sharply ≈300 °C and exhibits a slight increase under 650 °C. The stage below 300 °C corresponds to the evaporation of moisture in the sample, while the 300–650 °C stage corresponds to the combustion of the carbon layer. Under argon atmosphere, PVDF rapidly carbonizes at temperatures above 430 °C and provides a carbon source. As shown in Figure 2h, the final carbonized mass accounts for 25.1 wt.% of the total PVDF mass. The electrode ratio for the electrochemical lithiation reaction was Si: conductive carbon black: PVDF = 7:1.5:1.5. After electrochemical lithiation, high‐temperature CO_2_ reaction, and acid washing, the P‐Si@C material was obtained. Based on the TGA curve of P‐Si@C, its carbon content is 30.7 wt.%. Besides 14.85 wt.% from conductive carbon black, PVDF carbonization contributed 3.73 wt.% carbon, CO_2_ provided 12.12 wt.%, and silicon accounted for 69.3 wt.%. After excluding conductive carbon black, the mass ratio of silicon to carbon in P‐Si@C is 81.4:18.6.

As can be seen in Figure 2i,j, the surface of P‐Si@C exhibits a regular pore structure with uniformly distributed pores, which is markedly different from the smooth surface structure of pure Si (Figures S4 and S5, Supporting Information). To clearly observe the internal structure of P‐Si@C, we used a focused ion beam (FIB) to slice the sample. From the cross‐section of P‐Si@C (Figures 2k,l; S6, Supporting Information), it can be seen that numerous uniformly distributed pores are present, consistent with the BET results, further confirming the presence of a rich pore structure. This is attributed to the highly uniform Li_x_Si alloy resulting from electrochemical lithiation, which facilitates highly uniform oxide and carbon content in the intermediate product obtained by the subsequent reaction of Li_x_Si with CO_2_, thereby achieving uniform pores after acid washing. The rich pore structure provides space for expansion to alleviate Si expansion, promote electrolyte penetration, and enhance ion transport efficiency.^[^ ^22^ ^]^ To analyze the surface element distribution characteristics of the P‐Si@C, we performed energy‐dispersive X‐ray spectroscopy (EDS) detection on the surface and cross‐section. It can be seen from Figures 2m and S7 (Supporting Information) that Si and C elements are evenly distributed in P‐Si@C, indicating good uniformity of electrochemical lithiation, sufficient reaction with CO_2_, and uniform distribution of carbon layers. As shown in Figure 2n,o, it can be observed that the alternating light and dark patterns are caused by the pore structure, which is consistent with the SEM results. Figures 2p and S8 (Supporting Information) shows regular lattice stripes with a lattice spacing of ≈0.31 nm, corresponding to the (111) lattice spacing of Si.^[^ ^23^ ^]^ It is worth noting that a uniform carbon layer with a thickness of ≈3 nm can be observed on the outer layer of Si. Based on a comprehensive analysis of the results of Raman spectra, XPS, EDS, TGA, and HRTEMD, it can be concluded that the material has a rich and uniform carbon layer. The design of pore structure can effectively alleviate the huge volume expansion of Si, and the carbon layer improves the electrical conductivity of the composite material.

**Figure 3:**
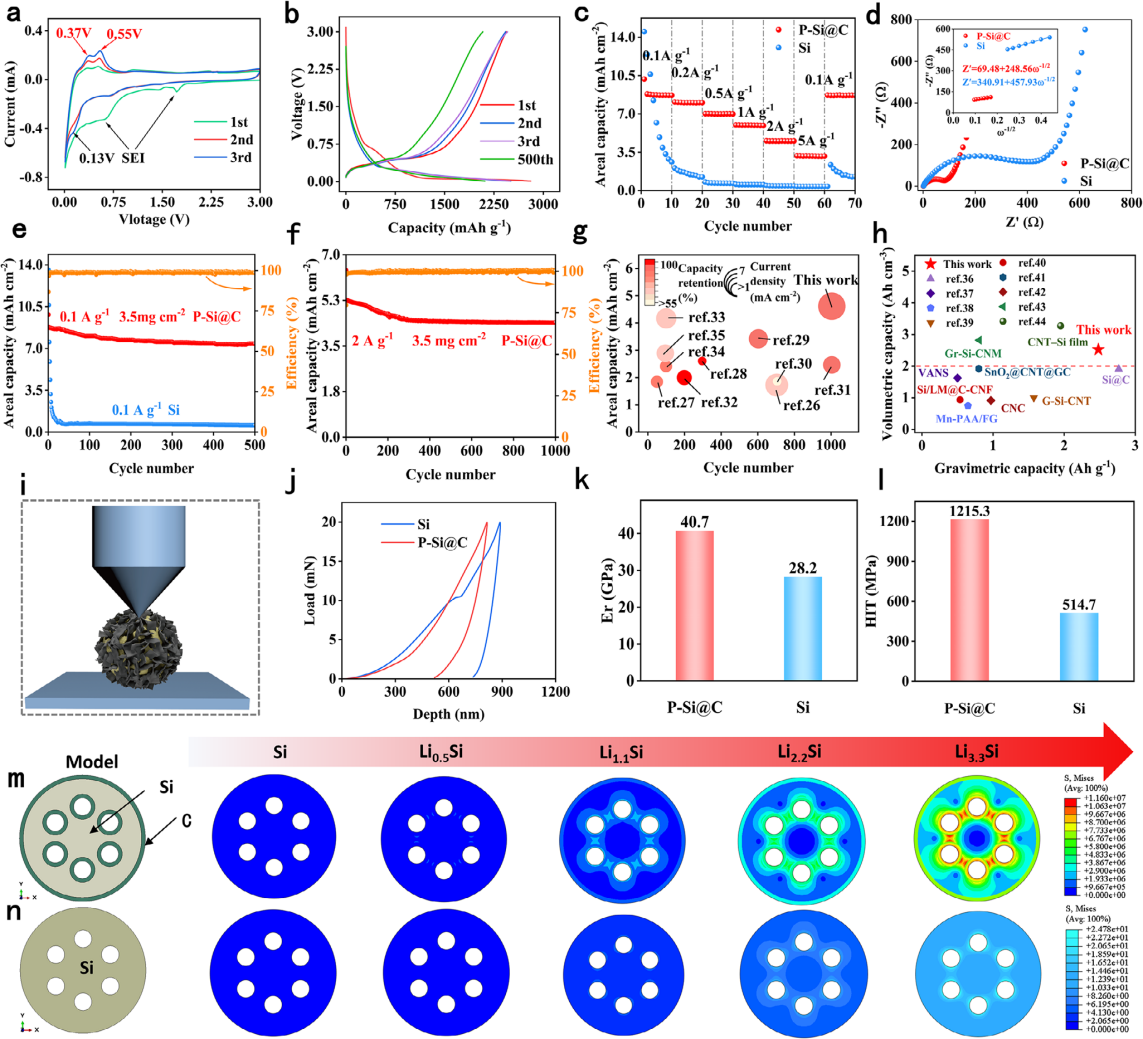
a–f) Electrochemical performance of LIBs with P‐Si@C as anode between 0.01–3 V. (a) CV curves of the first three cycles of P‐Si@C at a scanning rate of 0.1 mV s^−1^. (b) The first three and 500th cycle charge–discharge curves of P‐Si@C electrode at a current of 0.1 A g^−1^. (c) Rate performance of P‐Si@C and Si. (d) The EIS curves of P‐Si@C and Si, Z ' and b linear fitting curves of ω^−1/2^. (e) Cycle performance of P‐Si@C and Si electrode at 0.1 A g^−1^. (f) Cycle performance of P‐Si@C electrode at 2 A g^−1^. g) Comparison of electrochemical performance of Si‐based materials. h) Comparison of gravimetric and volumetric capacity of some typical anodes. i) Schematic diagram of nanoindentation test. j) Nanoindentation test curves of P‐Si@C and Si. k) Er of P‐Si@C and Si. 1) HIT of P‐Si@C and Si. m) Finite element model and lithiation simulation results of P‐Si@C. n) Finite element model and lithiation simulation results of porous Si.

**Figure** **3**a shows the first three cycles of the cyclic voltammetry (CV) curve for P‐Si@C at a scan rate of 0.1 mV s^−1^ and a voltage range of 0.01–3 V. It can be observed that the positive peaks (0.65 and 1.60 V) in the first cycle disappear in subsequent cycles, which is attributed to the formation of the SEI film.^[^ ^24^ ^]^ This positive peak (0.13 V) corresponds to Lithiation and the formation of a Li_x_Si alloy. During the oxidation process, two anode peaks (0.37 and 0.55 V) are associated with delithiation, resulting in the formation of amorphous Si. Figure 3b shows that the initial coulombic efficiency (ICE) of P‐Si@C reaches as high as 90.87%, significantly better than that of pure Si (78.83%). After multiple cycles, the cyclic Coulombic efficiency of P‐Si@C can reach as high as 99.9%. This is attributed to the sieve‐like porous structure, which not only promotes electrolyte diffusion, allowing lithium ions to pass quickly, but also provides a physical buffer for silicon expansion, significantly reducing the irreversible consumption of active lithium and electrolyte during the initial charge and discharge process. As can be seen in Figure 3c, the areal capacities of P‐Si@C electrodes are 8.7, 8.0, 7.0, 5.9, 4.5, and 3.2 mAh cm^−2^ (2493, 2296, 1998, 1704, 1294, and 901 mAh g^−1^) under current densities of 0.1, 0.2, 0.5, 1, 2, and 5 A g^−1^, respectively. When the current is restored to 0.1 A g^−1^, the areal capacity recovers to 8.7 mAh cm^−2^ (2485 mAh g^−1^), with a capacity retention rate of 99.7%. In contrast, pure Si electrodes exhibit rapid capacity decay at high current densities. To further investigate the reaction kinetics of the P‐Si@C electrode, electrochemical impedance spectroscopy (EIS) testing was performed. As shown in Figure 3d, the electrochemical impedance of the P‐Si@C electrode was only 69.48 Ω, significantly lower than that of pure silicon (340.91 Ω). The diffusion coefficient of lithium ions (D_Li+_) is one of the important indicators for measuring the diffusion rate of Li^+^ in electrodes. The calculated D_Li+_ for the P‐Si@C electrode is 1.03 × 10^−15^ cm^2^ s^−1^, significantly higher than that of the pure Si electrode (3.04 × 10^−16^ cm^2^ s^−1^). This further demonstrates that uniform carbon layer significantly enhances the conductivity of electrode, while the pore structure provides pathways for the rapid lithium ions diffusion. The formulas for calculating Z′ and D_Li+_ are explained in detail in the Supporting Information.^[^ ^25^ ^]^

As shown in Figure 3e, the P‐Si@C electrode showed high areal capacity and volumetric capacity of 7.4 mAh cm^−2^ and 2176 mAh cm^−3^, respectively, after the 500th cycle under 0.1 A g^−1^, with a capacity retention rate of 85.2%. We assembled w‐Si@C into half‐cells for cycling tests (Figure S9, Supporting Information), revealing that its performance was inferior to P‐Si@C but superior to pure silicon electrodes. Notably, the areal capacity of P‐Si@C electrode remains as high as 4.4 mAh cm^−2^ over 1000 cycles under 2 A g^−1^ with a capacity retention rate of 84.1% (Figure 3f). The P‐Si@C electrode demonstrates high areal capacity, excellent cycling stability, and fast charging/discharging performance, which significantly outperform previously reported Si‐based composite electrodes^[^ ^26^, ^27^, ^28^, ^29^, ^30^, ^31^, ^32^, ^33^, ^34^, ^35^ ^]^ (Figure 3g; Table S1, Supporting Information). This is attributed to the skeletal structure formed by the sieving pores, which provides buffer space for volume changes and maintains the overall integrity of the structure, while the nanopore network is embedded within or distributed on the surface of the skeleton, optimizing ion transport and charge transfer. The specific capacity and volumetric capacity of electrode materials jointly determine the energy storage efficiency of batteries, which are key indicators for evaluating battery performance and meeting the requirements of lightweight or miniaturized applications. The P‐Si@C electrode exhibits high specific capacity and volumetric capacity of up to 2480 mAh g^−1^ and 2523 mAh cm^−3^, respectively, which is significantly superior to others reported representative anodes in previous work, including carbon‐based, Si‐based, and Sn‐based materials (Figure 3h; Table S2, Supporting Information).^[^ ^36^, ^37^, ^38^, ^39^, ^40^, ^41^, ^42^, ^43^, ^44^ ^]^ The Volumetric capacity of the two materials studied, Gr‐Si‐CNM and CNT‐Si film, showed high volumetric capacities but modest gravimetric capacities. More importantly, the thin‐film structure has a low areal loading, resulting in low areal capacities, which is not conducive to practical applications. Furthermore, the preparation conditions were very stringent, resulting in silicon films, which are difficult to produce on a large scale. In contrast, the P‐Si@C preparation method used in this study is simpler and exhibits superior overall performance. To elucidate the mechanisms by which the carbon layer and pores enhance mechanical strength and mitigate volume expansion, we conducted nanoindentation testing and simulation analysis. As shown in Figure 3i–l, the Young's modulus (Er) and hardness (HIT) of P‐Si@C are 40.7 GPa and 1215 MPa, respectively, which are significantly better than those of Si (28.2 GPa and 515 MPa), representing improvements of 44.7% and 135.9%. We used Abaqus 6.14 finite element method (FEM) to establish simplified P‐Si@C and porous Si models, which simulated the stress distribution during lithiation,^[^ ^45^ ^]^ as shown in Figure 3m,n. As lithium is embedded under the same silicon and carbon simulation parameters, the Si gradually expands and generates internal stress. After mesh independence verification, the simulation results exhibit that the maximum internal stress of P‐Si@C model is 11.6 MPa, while the maximum internal stress of the porous Si model is only 25 Pa. Furthermore, the maximum strain of both P‐Si@C and porous Si was located at the outer layer, and the strain of P‐Si@C was lower than that of porous Si (Figure S10, Supporting Information). This indicates that the coordinated action of the carbon layer and hierarchical pore structure enhanced the mechanical strength and alleviated the expansion effect, reducing damage to P‐Si@C during the cycling process, which is conducive to improving its cycling stability.

**Figure 4:**
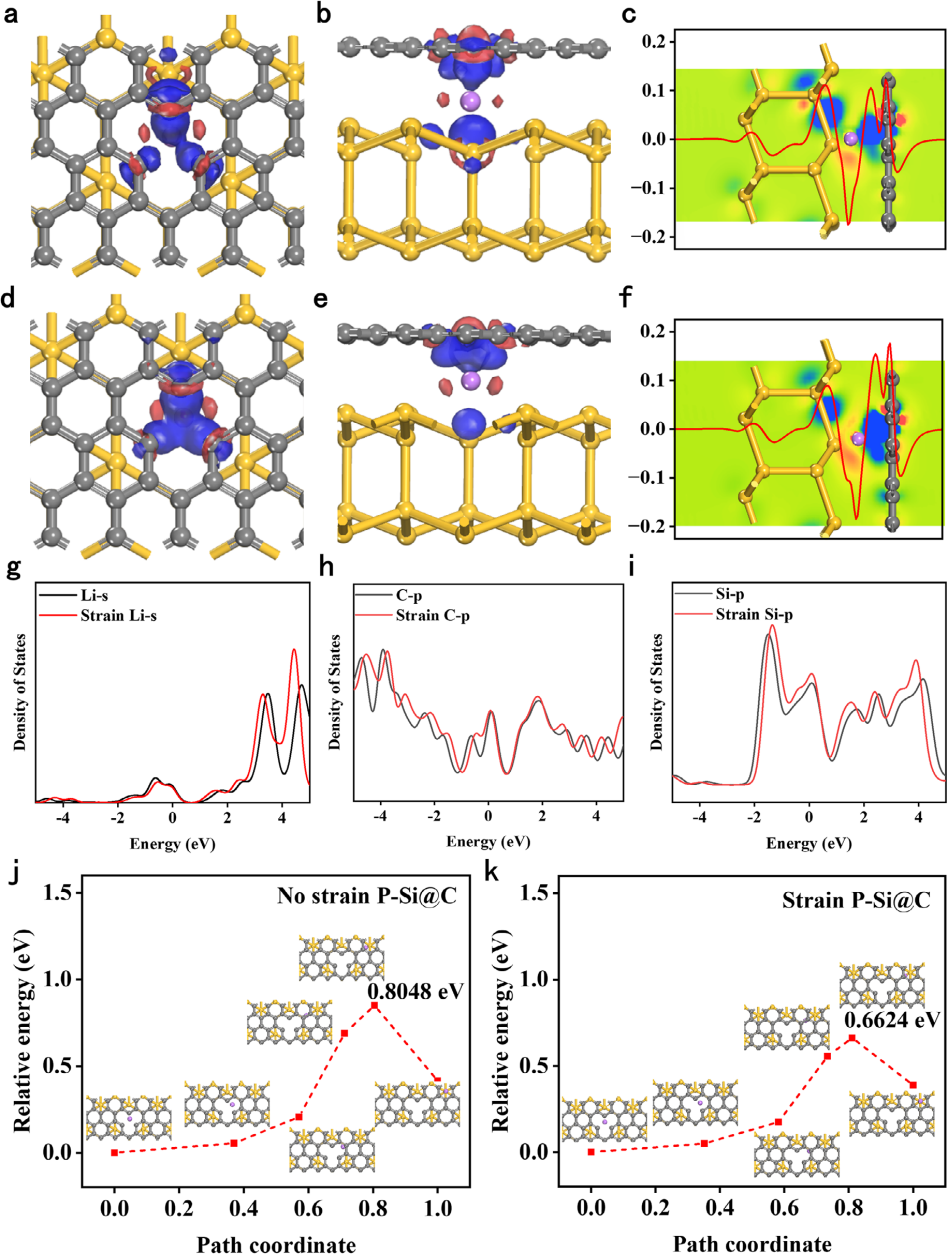
a) Top view and b) side view and c) charge density difference and charge accumulation curve of stress‐free P‐Si@C, with yellow and black spheres representing Si and C atoms, respectively. d) Top view and e) side view and f) charge density difference charge accumulation curve of the stressed P‐Si@C. Among them, the region where electrons accumulate is blue and the region where they are exhausted is red. Charge accumulation curve: the average value of electron density difference Δρ in *X*‐axis direction. g–i) Comparison of the density of states curves of P‐Si@C with and without stress, (g) density of states of Li‐s, (h) density of states of C–p, (i) density of states of Si–p. j) Ediff and migration paths of Li^+^ in P‐Si@C heterojunctions under stress‐free action. k) Ediff and migration paths of Li^+^ in P‐Si@C under stress.

To analyze the effect of stress on the electronic structure of P‐Si@C, we established stress‐free and stress‐induced P‐Si@C heterojunction models (**Figure** **4**a,b,d,e). Combining the results of nanoindentation testing with Abaqus simulations of P‐Si@C, it can be found that P‐Si@C generates significant internal stress during charging and discharging (Figure S11, Supporting Information). The difference in charge density between stressed and unstressed P‐Si@C indicates that stress affects the valence electron concentration between lithium ions and carbon atoms (Figure 4c,f). By comparing the charge accumulation curves, it can be observed that the charge between lithium ions and the carbon layer significantly increases due to stress, resulting in a more pronounced aggregation phenomenon. When stress is applied to the P‐Si@C electrode, the Li–s, Si–p, and C–p orbitals are significantly enhanced or displaced, which is mainly concentrated in the conduction band region (Figure 4g–i). The increase in the density of states indicates that the conductivity of the structure is enhanced. The stress response of P‐Si@C is very significant, which means that the conductivity of the material is significantly improved when stress is applied. The diffusion rate of Li^+^ in P‐Si@C is one of the key parameters influencing its electrochemical properties. To study the mechanism by which stress effects influence the diffusion properties of the material, we calculated the diffusion energy barrier (Ediff) of Li^+^ in P‐Si@C under both stressed and unstressed conditions, along with their corresponding ion migration paths. Among these, Ediff is the difference between the transition state energy and the stable structure energy. As shown in Figure 4j,k, the Ediff values away from the defect direction were found to be 0.8048 and 0.626 eV, respectively, through calculations on stress‐free and stress‐induced P‐Si@C. This indicates that stress reduces the Ediff value of Li^+^ and decreases the number of charge transfer events for Li, which weakens the bonding force between Si and Li^+^ and promotes the migration of Li^+^.^[^ ^46^ ^]^ Additionally, the Ediff values in the defect direction are significantly lower, indicating that Li^+^ tends to diffuse from the binding sites toward the defect direction, while stress does not alter the diffusion direction of Li^+^. The stress enhances the conductivity of P‐Si@C and reduces the Ediff value of Li^+^ in the material, which improves Li^+^ transport efficiency and enhances rate performance.^[^ ^47^ ^]^ Combining Abaqus simulation results, it was found that when lithium is embedded under carbon layer constraints, the internal stress distribution of P‐Si@C is greater than that of porous Si. This indicates that the Li^+^ diffusion energy barrier in P‐Si@C during Lithiation is lower, and the Li^+^ diffusion coefficient is larger, which is consistent with previous calculations of the diffusion coefficient.

**Figure 5:**
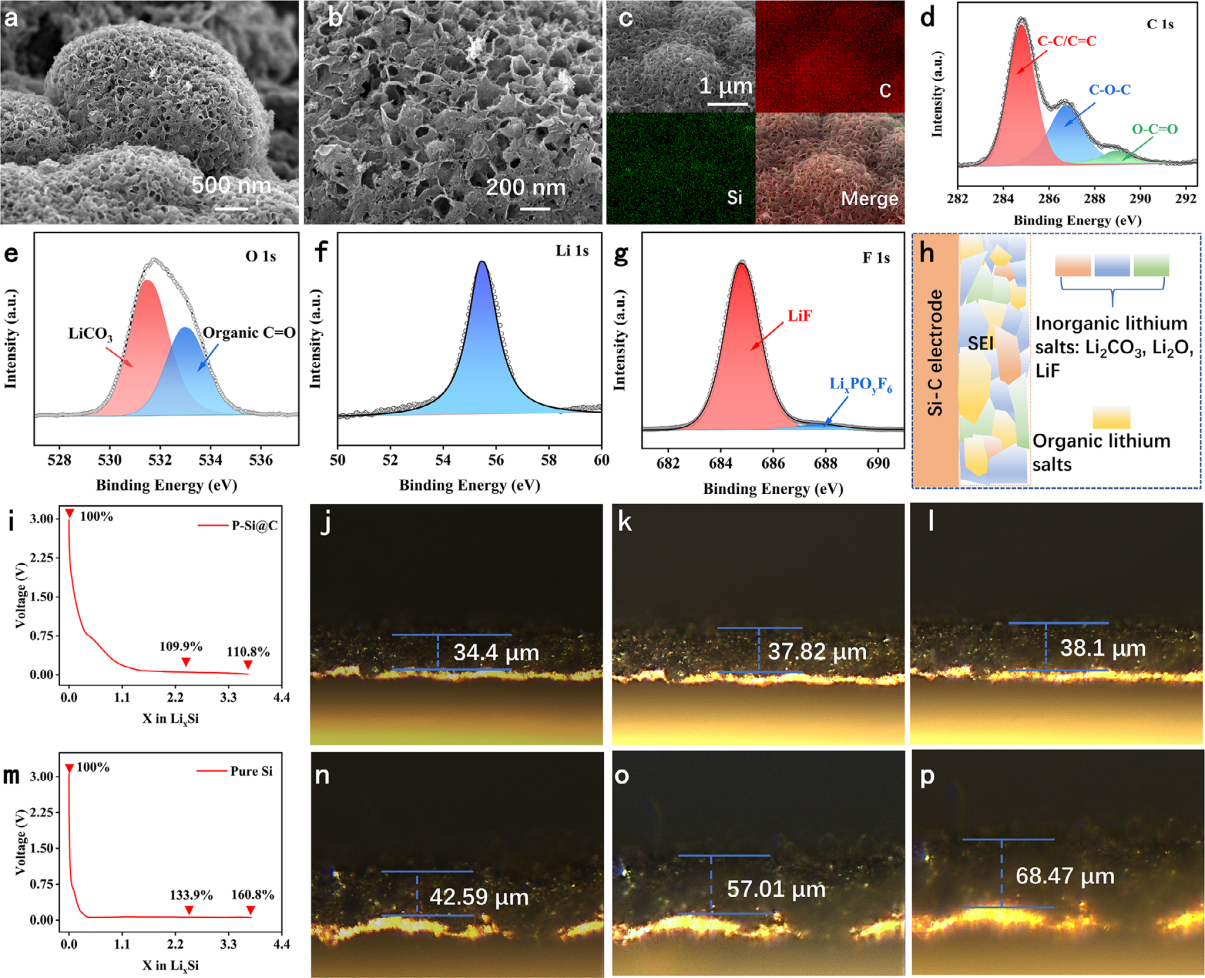
a,b) SEM images of P‐Si@C after 500 cycles. c) EDS mapping images of P‐Si@C after 500 cycles. d–g) XPS spectra of the electrode surface after 500 cycles, (d) C 1s, (e) O 1s, (f) Li 1s, (g) F 1s. h) Schematic diagram of surface SEI composition of P‐Si@C electrode after 500 cycles. i) The first discharge curve of P‐Si@C, j–l) Optical microscope images of electrode cross‐sections corresponding to different degrees of discharge. m) Discharge curve of the first cycle of pure Si. n–p) Optical microscope images of electrode cross‐sections corresponding to different discharge degrees of pure Si electrodes.

As shown in **Figure** **5**a–c, it can be observed that the morphology of the P‐Si@C remains intact and the Si and C elements are evenly distributed after 500 charge–discharge cycles, which indicates its excellent structural stability. We also performed XPS testing on the P‐Si@C electrode after 500 cycles to analyze the composition of the SEI film (Figure S12, Supporting Information). Among these, the C 1s spectrum can be decomposed into C─C/C═C (284.79 eV), C─O─C (284.74 eV), and O─C═O (288.89 eV) (Figure 5d).^[^ ^48^ ^]^ The O 1s spectrum primarily consists of two peaks at 531.44 and 532.97 eV, corresponding to Li_2_CO_3_ and organic C═O (Figure 5e).^[^ ^49^ ^]^ The peak position of the Li 1s spectrum is 55.43 eV (Figure 5f). The F 1s spectrum can be decomposed into two peaks at 684.75 and 687.67 eV, corresponding to LiF and Li_x_PO_y_F_6_ compounds (Figure 5g).^[^ ^50^ ^]^ It was found that LiF accounted for the majority of the peak, which was attributed to certain side reactions of salt decomposition.^[^ ^51^ ^]^ Therefore, the SEI film formed after charge–discharge cycles in P‐Si@C was mainly composed of lithium salts, namely inorganic lithium salts Li_2_CO_3_, LiF, Li_2_O, Li_x_PO_y_F_6_, and some organic lithium salts (Figure 5h).^[^ ^45^ ^]^ This indicates that the inorganic lithium salts and organic lithium salts in the SEI film are relatively balanced. The organic‐rich SEI film can buffer the changes of electrode volume, while the inorganic‐rich SEI film exhibits excellent ionic conductivity. The balanced SEI film can ensure the stability of battery cycling while maintaining good rate performance. To gain an intuitive understanding of the actual expansion behavior of P‐Si@C electrodes and the material suppression mechanism, we employed in situ optical microscopy technology to observe the electrode expansion phenomenon in real time. As shown in Figure 5m–p, the pure Si electrode undergoes a severe expansion of 160.8% during discharge, indicating that a large amount of Li+ insertion causes significant volume changes. In contrast, the thickness of the P‐Si@C electrode increased to only 110.8% of its original value, with a significantly reduced expansion rate, indicating excellent stress relief capability (Figure 5i–l). This is attributed to the synergistic effect of the hierarchical porous structure and uniform carbon layer, which effectively buffers Li^+^ insertion stress, suppresses volume changes, and significantly alleviates electrode deformation, thereby enhancing its electrochemical performance.

**Figure 6:**
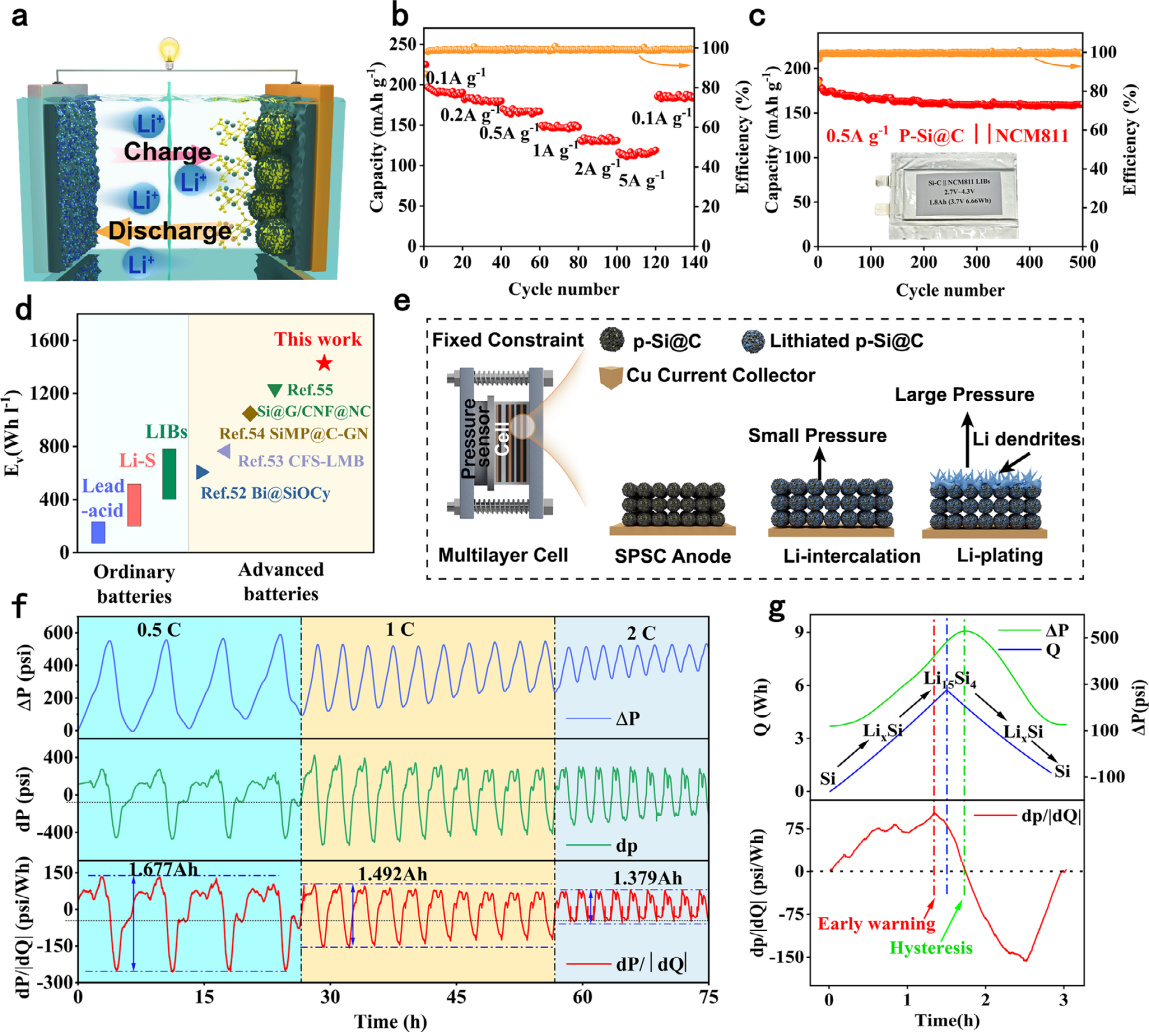
a) Schematic diagram of NCM811//P‐Si@C full cell. b) Rate performance of NCM811//P‐Si@C full cell. c) Cycle performance of NCM811//P‐Si@C full cell at 0.5 A g^−1^. d) Comparison of volumetric energy of NCM811//P‐Si@C full cell with the reported full cells. e) Schematic diagram of pressure monitoring for NCM811//P‐Si@C full cell full battery. f) Pressure monitoring data of cycles under different currents. g) The curve of pressure and charge.

To evaluate its practical application value, we assembled the P‐Si@C anode and NCM811 cathode into a full‐cell for performance testing (N/P = 1.08), where NCM811 is a commercial material (**Figure** **6**a), and also assembled it into a pouch cell for pressure monitoring. As shown in Figure 6b, the specific capacity of NCM811//P‐Si@C full cell reaches 192.6, 180.7, 166.9, 147.8, and 131.4 mAh g^−1^ at current densities of 0.1, 0.2, 0.5, 1, and 2 A g^−1^, respectively. Even at a high current density of 5 A g^−1^, the specific capacity of NCM811// P‐Si@C full cell remains as high as 114.7 mAh g^−1^. Meanwhile, when the current density is reduced to 0.1 A g^−1^, the specific capacity of the NCM811// P‐Si@C full cell rapidly recovers to 184.8 mAh g^−1^, indicating that the battery possesses excellent fast‐charging performance. After 500 cycles at a current density of 0.5 A g^−1^, the NCM811// P‐Si@C full cell achieved a capacity of 159.8 mAh g^−1^ with a capacity retention rate of 91.5%, demonstrating excellent cycling stability (Figure 6c). The charge and discharge curves of the full battery are stable with a relatively small degree of attenuation (Figure S13, Supporting Information). Unlike half‐cells, the voltage range of full cells is 2.7 to 4.3 V. The volumetric energy density of a battery is one of the core metrics determining its practical application performance, which determines its range capability within a limited space, thereby influencing the device's portability, runtime, and overall design flexibility. In this battery structure, the areal capacity of the electrodes is 4.34 mAh cm^−2^, and the battery thickness (including the anode, cathode, current collector, and separator) is ≈112.4 µm (Figure S14a, Supporting Information). The estimated volumetric energy density of this battery is 1428 W h L^−1^ (Figure S14b, Supporting Information). This value is nearly double that of commercially available NCM811//graphite batteries (640 W h L^−1^) calculated using the same metrics. It should be noted that this value is nearly double that of commercial NCM811//graphite batteries (640 W h L^−1^) calculated based on the same metrics, which is significantly superior to representative batteries reported (Figure 6d).^[^ ^52^, ^53^, ^54^, ^55^ ^]^ This demonstrates the feasibility of the P‐Si@C designed in this work as an anode for lithium‐ion batteries with ultra‐high volumetric energy density.

Real‐time monitoring of the expansion evolution of the internal structure of Si‐based batteries is key to accurately assessing their cycle life, which is crucial to ensuring the safety, reliability, and long‐term performance of batteries in practical applications.^[^ ^56^, ^57^ ^]^ To this end, we use pressure sensors to monitor the battery and establish reliable and accurate early warning signals for the battery based on the pressure data (Figure 6e). The pouch cell is composed of two layers of P‐Si@C anode, separator and NCM811 cathode, stacked in a bag made of aluminum laminated film, with a designed capacity of 1.8Ah (6.66 Wh). When manufacturing pouch batteries, space is reserved on the sides for gas expansion to ensure the accuracy of pressure information. The expansion caused during battery cycling can be divided into reversible expansion and irreversible expansion. The formation of the SEI film or the growth of lithium dendrites can lead to irreversible expansion. During charging and discharging, the battery undergoes reversible expansion due to the insertion and removal of lithium, while the positive electrode undergoes negligible volume change.^[^ ^58^ ^]^ It can be seen that the battery pressure curve fluctuates regularly with the charge–discharge cycle mode, with pressure increasing during charging and decreasing during discharge (Figure 6f). For the rate of pressure change, we use the derivative of pressure change (dp), which remains stable under the same current. To investigate the characteristics of the pressure signal, we convert the signal to dp/|dQ|. The average capacity of full battery is 1.677, 1.492, and 1.379 Ah at 0.5, 1, and 2 C, respectively. As the current increases, the capacity decreases, and the upper and lower limits of dp/|dQ| also decrease accordingly. As shown in Figure 6g, the peak of Δp occurs later than the peak of Q, while the peak of dp/|dQ| occurs before Q. The actual peak of battery charging is basically consistent with the peak of Q, while the peak of battery voltage lags behind Q. Therefore, we innovatively propose the peak point of dp/|dQ| as a warning signal, which is combined with the voltage signal to regulate the battery charging status. When the peak value of dp/|dQ| is reached, the system issues a warning and switches to low‐current charging mode to prevent the formation of lithium dendrites and overcharging.

**Figure 7:**
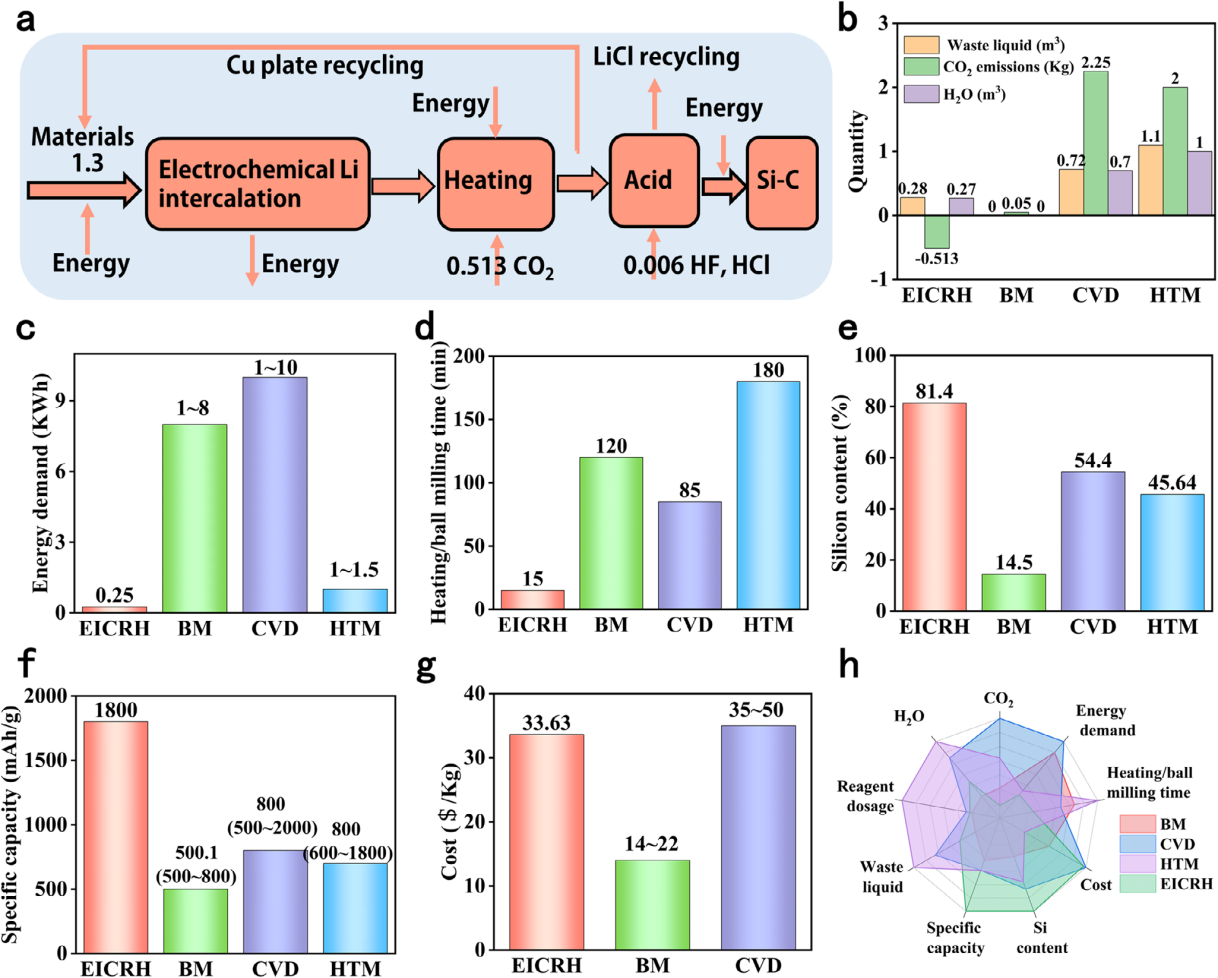
Economic and environmental friendliness assessment. a) EICRH raw material flow chart, the numbers in the figure represent the flow rate of substances, with the unit being kilograms. b) The amount of waste liquid and CO_2_ produced, as well as water consumption. c) Energy demand. d) Heating/ball milling time. e) Si content. f) Specific capacity. g) Cost. h) Comprehensive comparison of different methods.

To assess the economic and environmental friendliness of P‐Si@C prepared using the innovative fusion of electrochemical intercalation and CO_2_ rapid heating technology (EICRH), we calculated the raw materials and energy consumption required to prepare 1 kg of P‐Si@C (**Figure** **7**a; Tables S3 and S4, Supporting Information). The EICRH preparation route was compared with silicon–carbon composites prepared via ball milling (BM), chemical vapor deposition (CVD), heat treatment method (HTM).^[^ ^59^, ^60^, ^61^ ^]^ The silicon‐carbon materials prepared by BM and CVD have already been applied in the market. Therefore, the cost range of the BM and CVD methods is estimated based on the market. In terms of CO_2_ emissions, EICRH can absorb carbon dioxide as a carbon reduction technology, which can promote the resource utilization and high‐value conversion of CO_2_ (Figure 7b). In addition, the amount of waste liquid and water consumption is also not high. In terms of energy consumption, EICRH has the lowest energy consumption, which is 50% lower than that of the traditional CVD method (Figure 7c). This is attributed to the short heating time, ultra‐fast heating rate, and excellent heating efficiency of the electromagnetic induction heating method. In addition, EICRH demonstrates significant advantages in key performance metrics such as preparation efficiency, silicon content, and specific capacity (Figure 7d–f). After comprehensive comparative analysis, BM has the advantages of simplicity, environmental friendliness and low cost, but its capacity is relatively low. CVD is the opposite of BM, and HTM is not yet mature. EICRH integrates core advantages such as fast heating, high efficiency, carbon absorption and environmental friendliness (Figure 7h). A significant portion of the cost of EICRH is the raw material Li. During this process, Li does not completely become waste but turns into a LiCl solution containing very few impurities. The recycling and reuse of LiCl solution can significantly reduce costs. This not only provides an advanced solution for the large‐scale production of P‐Si@C that combines outstanding economic viability with exceptional environmental friendliness, but also opens up an innovative path for high‐energy batteries to develop in a low‐carbon and sustainable direction through its unique “negative carbon” potential.

## 3 Conclusion

In summary, we innovatively combined electrochemical lithiation with CO_2_ rapid heating reaction technology to prepare high‐performance sieve‐pore silicon–carbon composite anodes (P‐Si@C) from photovoltaic waste silicon. This strategy achieves the mechanical constraint of Si within the pores through pore structure regulation, generating a stress‐voltage coupling effect, and significantly improving the kinetics and structural stability of the Si (de)alloying reaction. The P‐Si@C electrode exhibits a high capacity and low expansion rate (10.8% at 2493 mAh g^−1^ and 8.72 mAh cm^−2^), excellent rate performance (1257 mAh g^−1^ and 4.4 mAh cm^−2^ at 2A after 1000 cycles), and ultra‐low capacity decay (0.016% per cycle), which exceeds others reported Si‐based batteries in previous work. The full cell achieves a capacity retention rate of 91.5% over 500 cycles and a record‐breaking volumetric energy density of 1428 Wh L^−1^. Combining in situ microscopic observation, simulation, and DFT calculations revealed the correlation mechanism between material stress and electrochemical performance. A novel method was proposed for real‐time monitoring of expansion pressure using a pressure sensor, with the peak of dp/|dQ| serving as a failure warning signal for Si‐based battery safety. This work not only resolves the core contradiction of Si anodes through sieve structure design and stress control mechanisms, but also provides an efficient and environmentally friendly solution for the resource utilization of photovoltaic Si waste.

## Conflict of Interest

The authors declare no conflict of interest.

## Author Contributions

Y.W. contributed to the conceptualization, investigation, formal analysis, and methodology of the study, and prepared the original draft of the manuscript. R.W. contributed to the conceptualization, investigation, formal analysis, and methodology of the study. S.L. and H.L. contributed to data curation, investigation, and methodology, while R.M. provided supervision, conducted formal analysis, and oversaw writing—review and editing. Correspondence was addressed to R.M., and all authors revised the article and approved its final version.

## Supporting information



Supporting Information

## Data Availability

The data that support the findings of this study are available from the corresponding author upon reasonable request.
