# Supplementary material for: Tailoring Sieving Pores and Electrochemical Interface Intercalation for Mechanically Resilient Recycled Micro‐Silicon Anodes
Source: Adv Sci (Weinh). 2025 Dec 5;13(11):e17656. doi: 10.1002/advs.202517656 (PMC12931176; doi:10.1002/advs.202517656)
Supplement: Supplementary file 1 — Supporting Information [file ADVS-13-e17656-s001.docx]

**Tailoring Sieving Pores and Electrochemical Interface Intercalation for Mechanically Resilient Recycled Micro-Silicon Anodes**

Yunan Wei^a^, Ruilin Wu^a^, Han Liang^a^, Shixin Liu^a^, Runwei Mo^a,b*^

^a^ School of Mechanical and Power Engineering, East China University of Science and Technology, Shanghai 200037, China

^b^ Shanghai Key Laboratory of Intelligent Sensing and Detection Technology, East China University of Science and Technology, Shanghai 200237, China

∗Corresponding authors.
E-mail address: morunwei@126.com (R.W. Mo)

**Support Information**

**Experimental Section**

**1 Synthesis of** **Sieving-Pore Silicon/Carbon Composite**

The collected photovoltaic waste Si material is ground to micron level, mixed in a ratio of Si: conductive carbon black: PVDF = 7:1.5:1.5, and coated onto copper plates to form silicon electrodes. An electrolytic cell was assembled in an argon atmosphere glove box using a silicon electrode as the working electrode and a lithium foil as the counter electrode. The electrolyte was 1 M lithium hexafluorophosphate (LiPF_6_), a mixture of ethylene carbonate (EC)and Dimethyl carbonate (DMC) (1:1) with a 5% ethyl methyl carbonate (EMC) additive. The volume of the electrolyte added to the electrolytic cell is approximately 107.8 cm^3^. Electrochemical lithiation was performed in the electrolytic cell by discharging to 0.01 V to obtain Li_x_Si alloy. Subsequently, the Li_x_Si sample was transferred to an electromagnetic induction furnace under argon protection and heated at a power of 300 W (15.8 A, 19 V) for 5 minutes to dissolve the remaining electrolyte on the alloy surface. Then the power was increased to 500W (21.7A, 23V), and carbon dioxide was introduced to react thoroughly with Li_x_Si. Due to rapid heating with CO_2_, Li is converted into Li_2_O. PVDF carbonizes due to high temperatures. After the reaction is complete, the prepared product is placed in dilute hydrochloric acid with a concentration of 1.5 mol/L and subjected to ultrasonic treatment to ensure that the product reacts fully with the hydrochloric acid to remove Li_2_O or other oxides. The product is then acid-washed with a 1.5 mol/L HF solution to remove any SiO_x_. Pickling to remove oxides forms a rich sieve-like pore structure in the material. Finally, the material is washed with deionized water and ethanol, followed by vacuum filtration, to obtain sieving-pore silicon/carbon composite (P-Si@C), which is vacuum-dried under 100 °C for 12 h and stored for later use.

**2 Material Characterization**

We used an X-ray powder diffractometer (Rigaku Corporation Smart Lab 9KW), an X-ray photoelectron spectrometer (Thermo Fisher Scientific ESCALAB 250Xi), and a Raman spectrometer (Horiba LABRAM HR Evolution, 532 nm laser) to detect the crystal phase, chemical bonds, and chemical composition of the material, respectively. The Brunner-Emmert-Taylor (BET) specific surface area test was performed using Micromeritics 3Flex to test the specific surface area and porosity. Field emission scanning electron microscope (FESEM, Carl Zeiss Gemini SEM 500), focused ion beam scanning electron microscope (FIB-SEM, TESCAN LYRA3), transmission electron microscope (TEM), and high-resolution transmission electron microscope (HRTEM) were used to observe the microstructure and cross-section of the samples (JEM-2100). We used a thermogravimetric analyzer (PerkinElmer TGA 8000) to test the carbon content and thermal stability of the P-Si@C sample in an air atmosphere, and the PVDF was carbonized in an argon atmosphere. The Young's modulus and hardness of samples were measured using the nanoindentation tester (Anton Paar CPX-NHT2). An optical microscope (Yuescope, YM710TR) was used to observe the cross-section of electrodes *in situ* during charging and discharging, which observed changes in the thickness of electrode cross-sections made of different materials. The pressure sensor adopted is RP-C18.3 from Shenzhen Jinke Electronic Technology Co., LTD., and the monitoring equipment used is KEYSIGHT-34465A multi-functional digital multimeter.

**3 Electrochemical Performance**

The button cell was tested using lithium metal as counter electrode and Celgard 2400 as separator. The electrode paste was uniformly mixed in a ratio of active material: conductive carbon black: SBR: CMC = 7:2:0.5:0.5, then coated onto copper foil to form the electrode, with a surface loading of approximately 3.5 mg cm^-2^. The 20% conductive carbon black used for electrode manufacturing includes the conductive carbon black already present in the synthesis P-Si@C. Subsequently, the electrodes were placed in a vacuum drying oven and vacuum dried under 100 °C for 12 h. The electrolyte was 1 M lithium hexafluorophosphate (LiPF_6_), a mixture of ethylene carbonate (EC)and Dimethyl carbonate (DMC) (1:1) with a 5% ethyl methyl carbonate (EMC) additive. The cathode material of the cell is NCM811, specifically LiNi_0.8_Co_0.1_Mn_0.1_O_2_. The cathode material composition is NCM811: conductive carbon black: PVDF = 7:2:1, with a loading mass of 22 mg cm^-2^. The cathode current collector is aluminum foil. The N/P ratio of the full battery is 1.08. The working voltage range of the full battery is 2.7~4.3V. The pouch cell is composed of two layers of P-Si@C anode, separator and NCM811 cathode, stacked in a bag made of aluminum laminated film, with a designed capacity of 1.8Ah (6.66Wh). The battery is assembled in an argon-protected glove box. Electrochemical performance was tested using the NEWARE high-precision battery performance testing system (CT-4008-5V 6A-164), and all batteries were subjected to constant current charge and discharge tests. We chose an electrochemical workstation (CHI 604f) for electrochemical impedance spectroscopy (EIS) and cyclic voltammetry (CV) testing. The CV scan rate was 0.1 mV s^-1^, with a working voltage range of 0.01~3 V. The electrochemical impedance spectroscopy (EIS) test frequency range was 0.01~100 kHz.

**4 Battery Pressure Monitoring**

When packaging pouch batteries, reserve side space to avoid the possible gas generated by the battery from affecting pressure monitoring. Separate the battery from the pressure sensor with a separator to ensure that the pressure on the battery is uniform. Then, the battery is fixed with a fixture and subjected to 0.5C, 1C, and 2C charge and discharge cycle tests (1C =1 A g^-1^), and the impedance changes of the sensor are monitored in real time. The impedance signal is converted into a pressure signal through the relationship between impedance (R) and pressure (P), $R=153.18P^{-0.699}$.

**5 Calculation Method**

The ion migration pathway and energy barrier were studied through Linear Synchronous Transit (LST) and Quadratic Synchronous Transit (QST) methods implemented in CASTEP. First, geometry optimizations were carried out for both the initial and final configurations of the migrating ion to obtain fully relaxed local minima. The structural optimizations were performed based on density functional theory (DFT) with the Perdew–Burke–Ernzerhof (PBE) functional within the generalized gradient approximation (GGA), using ultrasoft pseudopotentials to describe electron–ion interactions. A plane-wave cutoff energy of 500 eV and a k-point spacing of 0.04 Å^-1^ were employed. The optimized structures were then used as input for the LST method to estimate the transition state geometry along the ion migration path. This geometry was further refined using the QST method to accurately locate the transition state and determine the ion migration energy barrier. During all calculations, the total energy and force convergence thresholds were set to 1.0×10^-5^ eV/atom and 0.05 eV/Å, respectively. Supercells were constructed to accommodate sufficient migration distance. To eliminate interactions between periodic images, a vacuum layer of 15 Å was added.

**6 Statistical Analysis**

No formal statistical testing was applied, as the consistent performance trends across replicates are the primary indicator of material performance. To ensure the reported data are reproducible, electrochemical evaluations involving charge-discharge cycling were conducted with at least three separately fabricated cells (n ≥ 3) for each type of electrode.

The calculation formulas for Z′ (1) and D_Li+_ (2) are as follows.

$Z^{'}=R_{b}+R_{ct}+\sigma\omega^{-1/2}$ (1)

$D_{{Li}^{+}}=\frac{R^{2}T^{2}}{2A^{2}n^{4}F^{4}C^{2}\sigma^{2}}$ (2)

In the formula, σ is the slope determined by the linear fitting Z', R is the gas constant, T is the absolute temperature, A is the effective area of the electrode, n is the number of charged electrons, F is the Faraday constant, C is the concentration of lithium-ion.

**
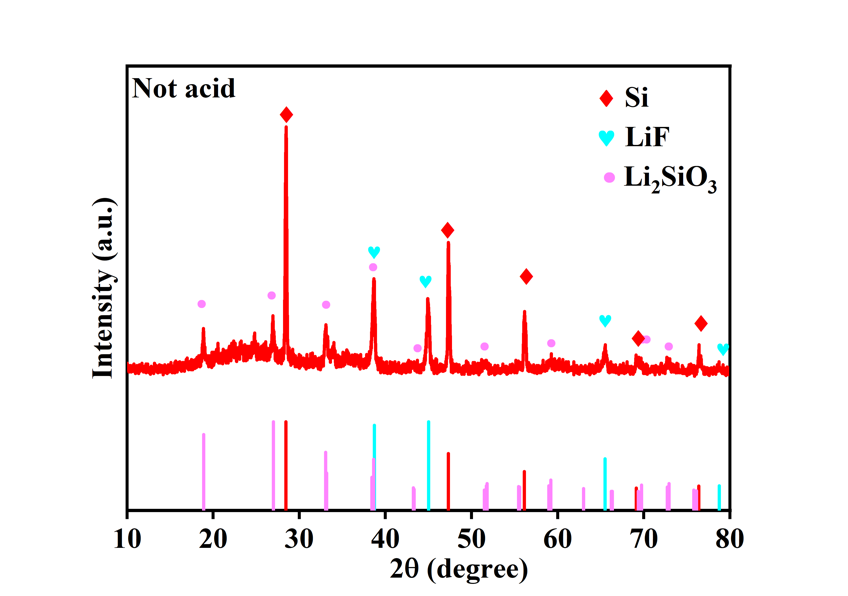
**

**Figure S1** XRD pattern of unpickled intermediate products.


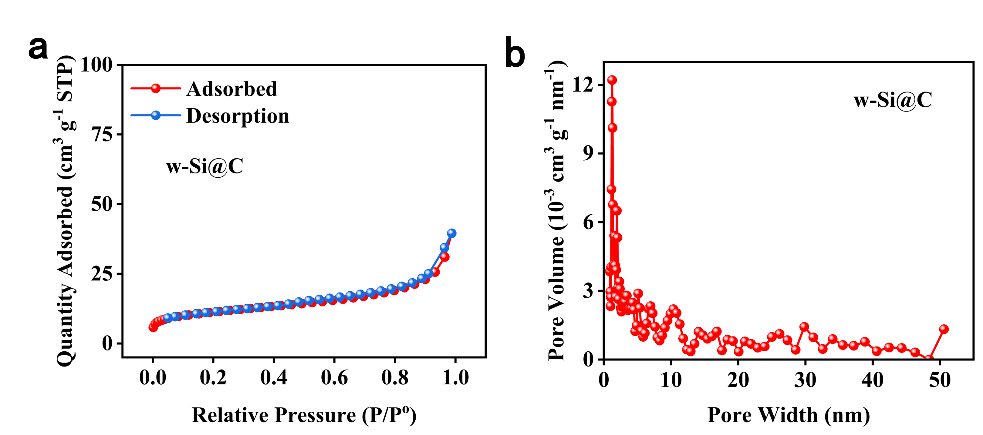


**Figure S2** (a-b) BET absorption-release curve and the pore size distribution of w-Si@C. w-Si@C is synthesized by heating electrode raw materials in the same ratio as P-Si@C, but without electrochemical lithiation and CO_2_ reaction.


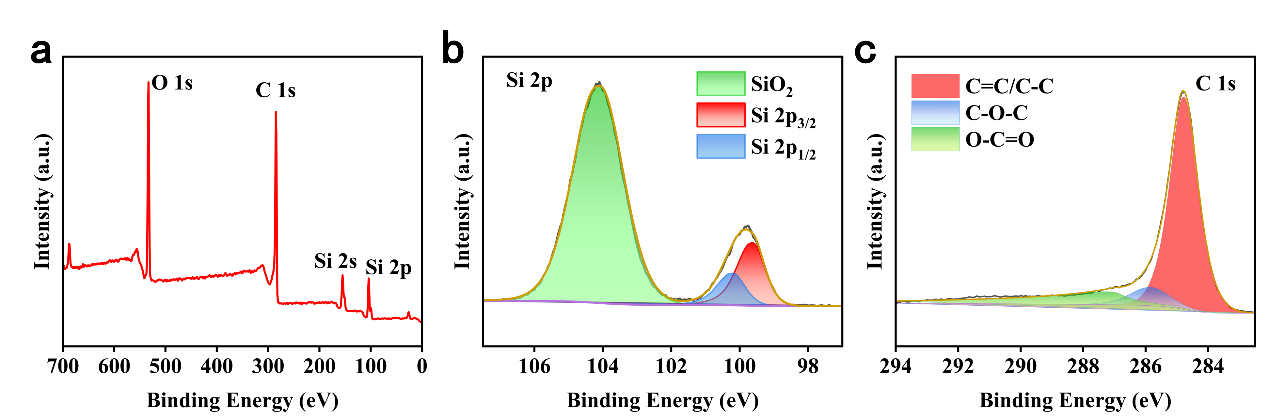


**Figure S3** (a) XPS spectra of w-Si@C, (b) C 1s, (c) Si 2p.

**
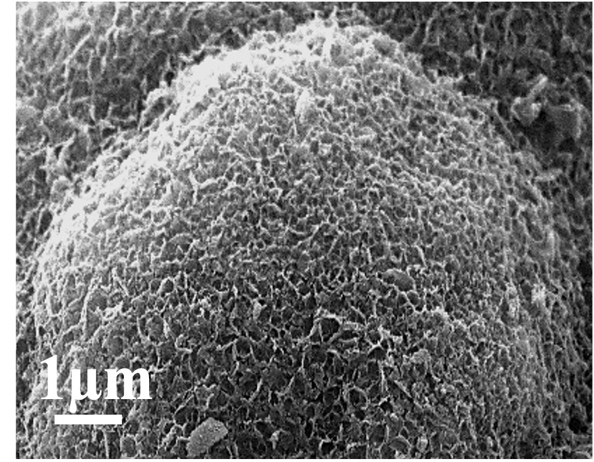
**

**Figure S4** SEM images of P-Si@C.


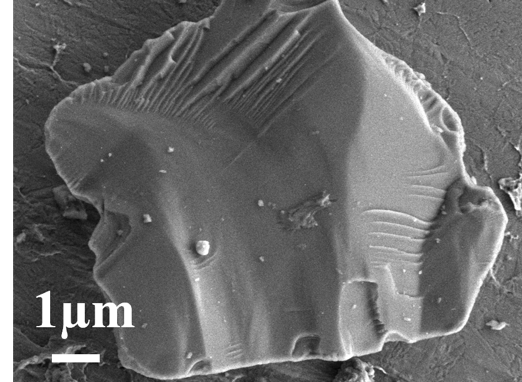

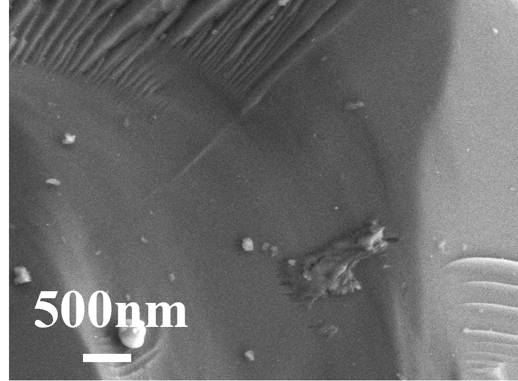


**Figure S5** SEM of photovoltaic waste Si material.

**
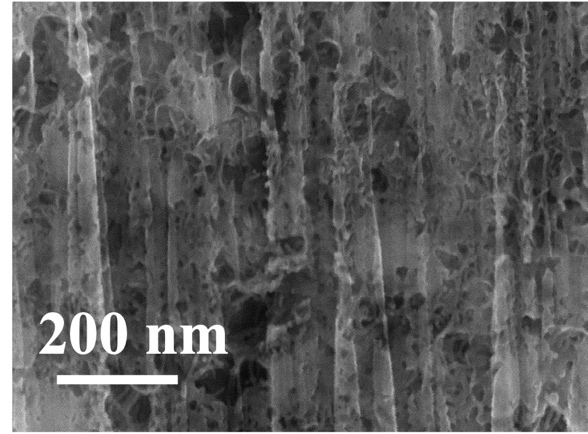
**

**Figure S6** SEM image of the cross section of P-Si@C obtained by FIB processing.

**
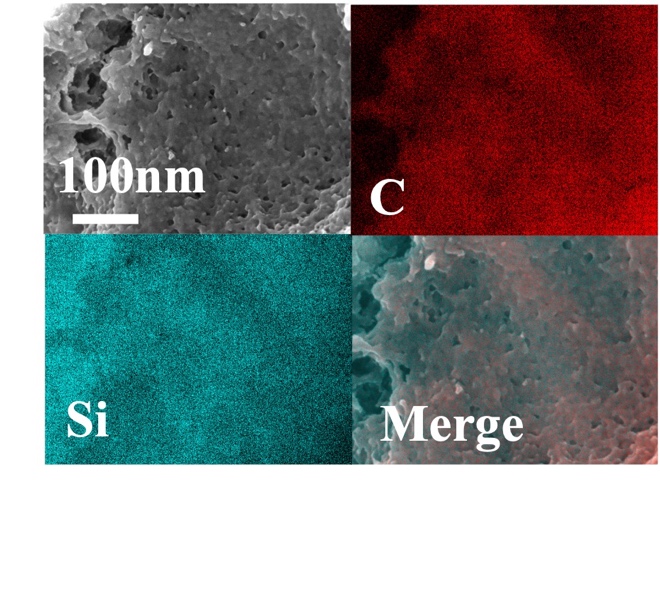
**

**Figure S7** EDS images of P-Si@C.


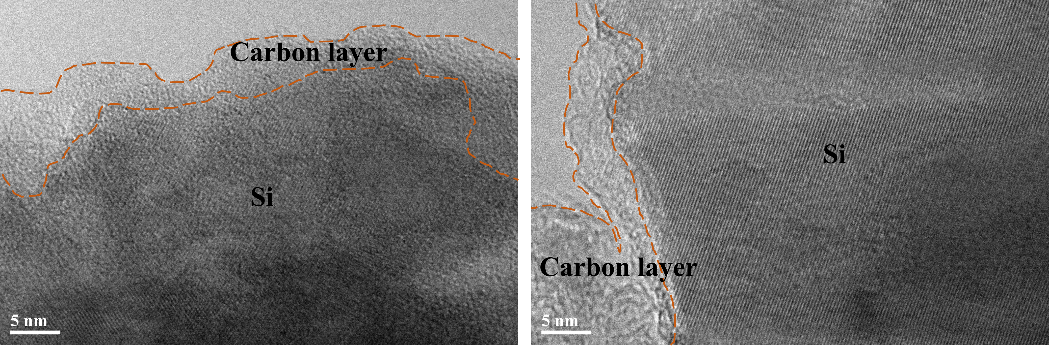


**Figure S8** HRTEM of P-Si@C


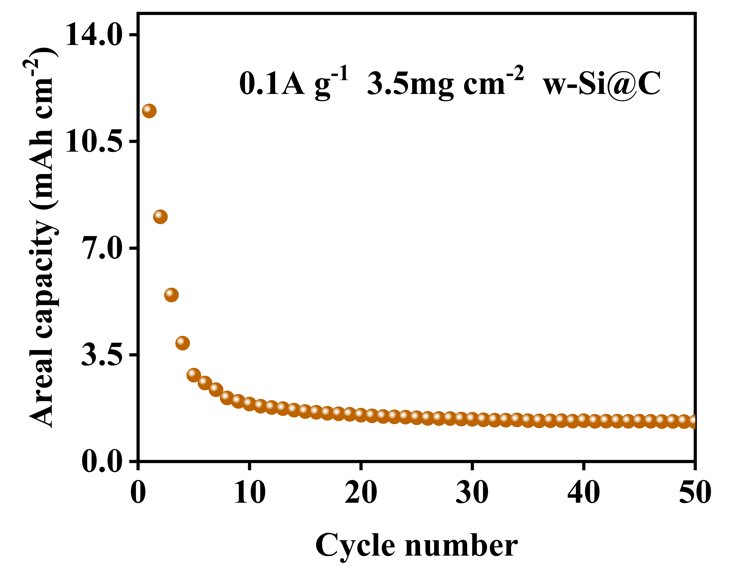


**Figure S9** Cycle performance of w-Si@C electrode at 0.1 A g^-1^.


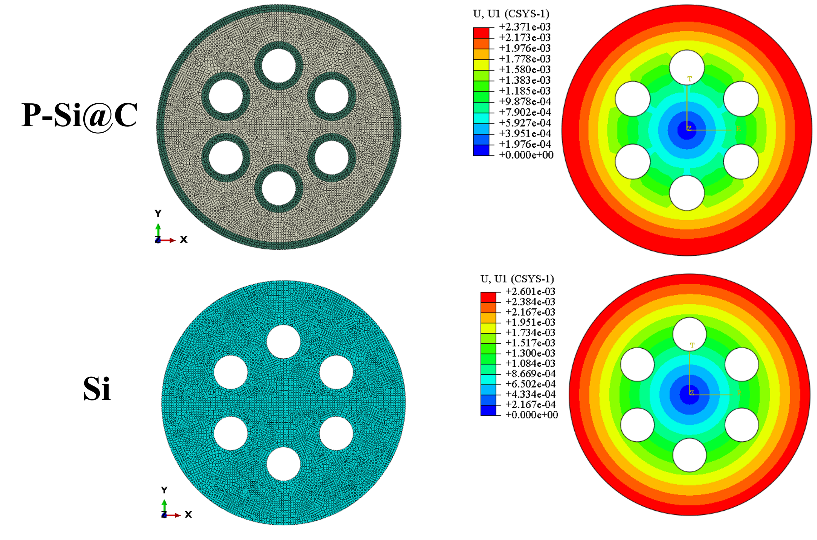


**Figure S10** Grid and strain distribution of Si and P-Si@C.


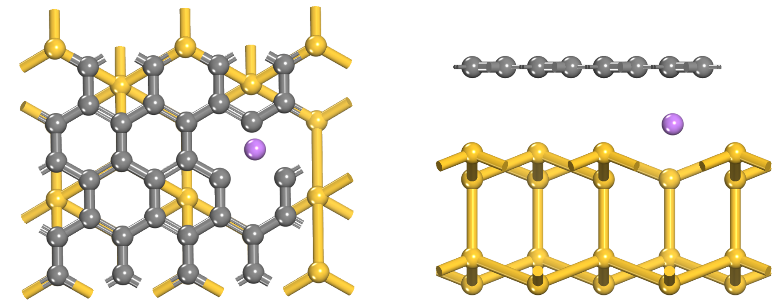


**Figure S11** Top view and front view of the basic model of P-Si@C.


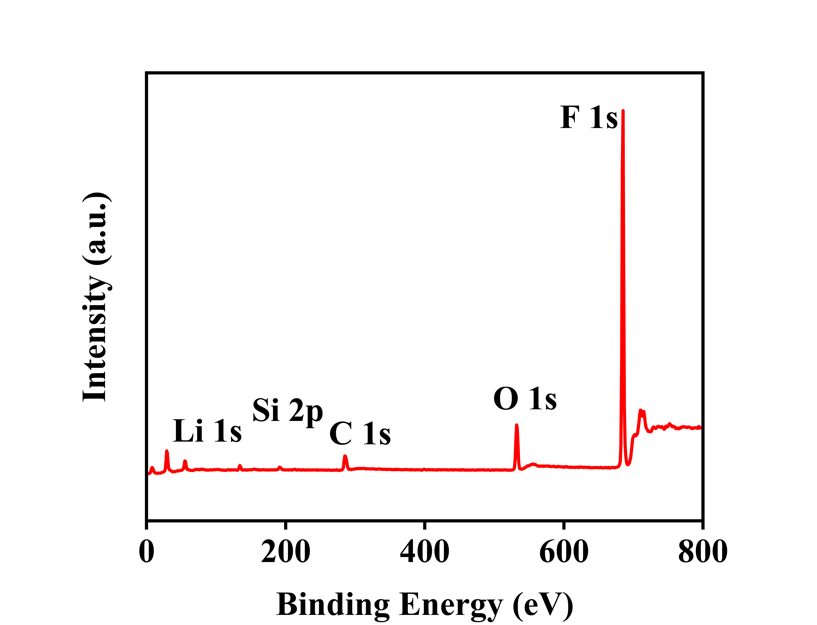


**Figure S12** XPS spectra of the P-Si@C electrode surface after 500 cycles.


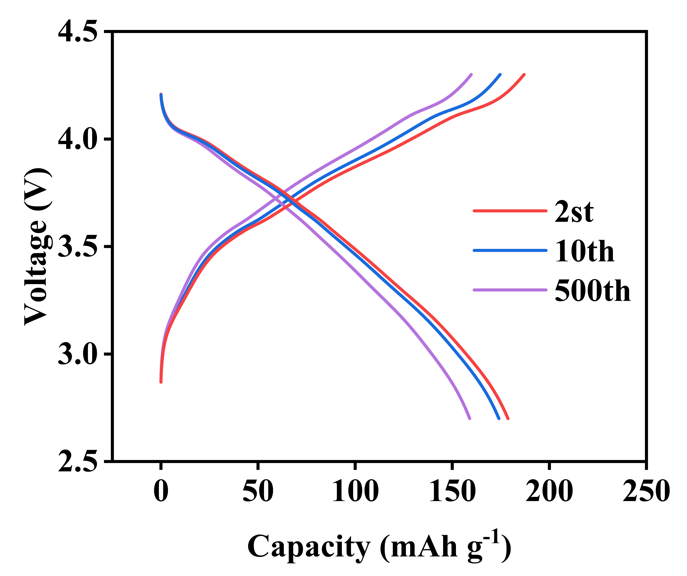


**Figure S13** charge-discharge curves of P-Si@C full cell at a current of 0.5 A g^-1^.


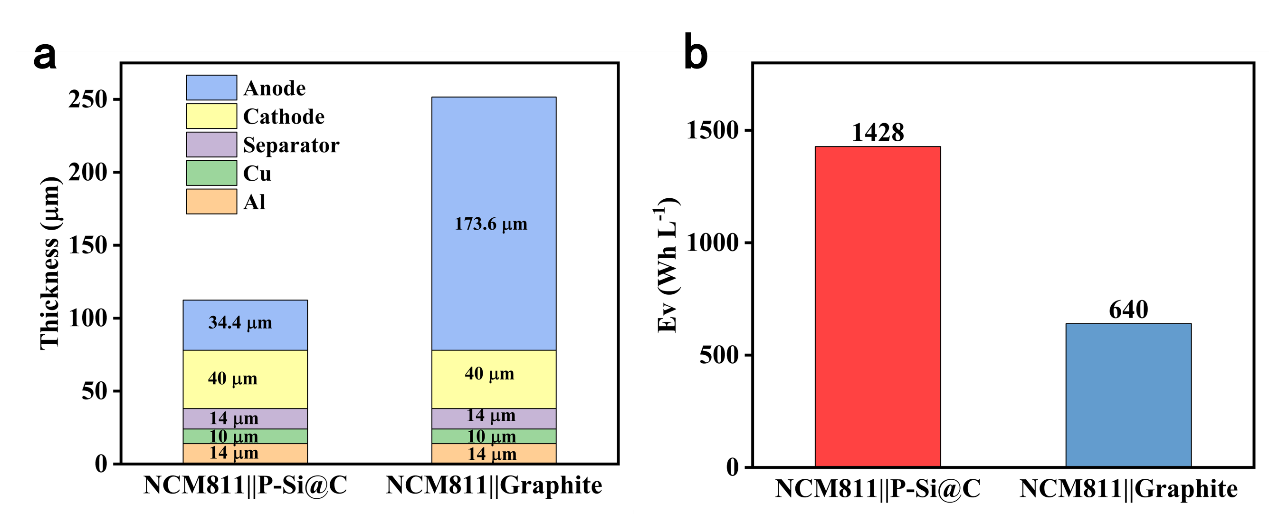


**Figure S14** (a) Full cell thickness of NCM811|| P-Si@C and NCM811||Graphite. (b) The volumetric energy density of NCM811|| P-Si@C and NCM811||Graphite. The thickness of NCM811|| P-Si@C and NCM811||Graphite is calculated under the same conditions, and this thickness is a comparison of the two types of batteries at the same capacity.

**Table S1** Comparison of electrochemical properties of typical Si-based anode and Si-C anode.

| Ref. | Names | Cycle number | Areal capacity (mAh cm^-2^) | Current density (mA cm^-2^) | Capacity retention (%) |
| --- | --- | --- | --- | --- | --- |
| This work | P-Si@C | 1000 | 4.4 | 7 | 84 |
| [1] | Si-SiC@C | 700 | 1.65 | 4.8 | 72 |
| [2] | Si@RFC-cPANi/G | 50 | 1.85 | 0.51 | 86 |
| [3] | pSi@void@NMC | 300 | 2.65 | 0.3 | 95 |
| [4] | SiNWs@CC | 600 | 3.2 | 2.01 | 83 |
| [5] | SP-MSi/C | 700 | 1.8 | 0.5 | 55 |
| [6] | VGSs-YS-Si/C | 1000 | 2.43 | 1.26 | 80 |
| [7] | SSC | 200 | 2 | 0.79 | 97 |
| [8] | Gr@Si@C | 100 | 4.2 | 3.75 | 72 |
| [9] | Reduced SiA/Ox-SWCNT/GO | 100 | 2.44 | 0.59 | 82 |
| [10] | p-mSi@SiOx/C | 100 | 2.87 | 1.1 | 72 |

**Table S2** Comparison of gravimetric and volumetric capacity of some typical anodes

| Ref. | Names | capacity (mAh g^-1^) | Volumetric capacity (mAh cm^-3^) |
| --- | --- | --- | --- |
| This work | P-Si@C | 2480 | 2523 |
| [11] | Si@C | 2767 | 1909 |
| [12] | VANS | 500 | 1625 |
| [13] | Mn-PAA/FG | 647 | 743 |
| [14] | G-Si-CNT | 1573 | 1006 |
| [15] | Si/LM@C-CNF | 536 | 936 |
| [16] | SnO_2_@CNT@GC | 800 | 1920 |
| [17] | CNC | 970 | 915 |
| [18] | Gr-Si-CNM | 806 | 2821 |
| [19] | CNT–Si film | 1950 | 3271 |

Table S3 provides detailed cost data for manufacturing 1 kg of P-Si@C via the EICRH method (excluding conductive carbon black), as conductive carbon black falls under battery manufacturing costs. After excluding conductive carbon black, the mass ratio of silicon to carbon in P-Si@C is 81.4:18.6. Carbon dioxide contributes 14.22% of the carbon element. Therefore, the value of x in equation (2) can be determined as 1.6, and the alloy can be expressed as Li_1.6_Si. The reference price is an estimated value subject to market fluctuations. Raw material requirements are calculated based on the electrochemical lithium-containing product Li_1.6_Si, with other material quantities also calculated against this benchmark. Energy consumption includes electricity usage for furnace operation, electromagnetic heating, and filtration processes. Given potential evaporation losses of electrolyte during electrode transfer, this component is also factored into costs. Considering the difficulty in achieving 100% material utilization and equipment wear factors, the total cost of $32.03/kg is multiplied by a factor of 1.05, resulting in an estimated cost of $33.63/kg. The majority of the costs stem from raw lithium materials. In this process, lithium is not entirely converted into waste but instead forms a lithium chloride solution with minimal impurities. Recycling and reusing the lithium chloride solution can significantly reduce costs.

**Table S3** Detailed data on the cost of preparing 1Kg P-Si@C by EICRH (excluding conductive carbon black)

|  | Energy consumption | 37% HF | 40% HCl | H_2_O | Waste Si | PVDF | Li | Electrolyte | Total cost (＄/Kg) |
| --- | --- | --- | --- | --- | --- | --- | --- | --- | --- |
| Reference price | 0.0492＄/KWh | 491.9＄/m^3^ | 21.1＄/m^3^ | 1.35＄/m^3^ | 2.1＄/Kg | 8.01＄/Kg | 81.5＄/kg | 2.8＄/Kg | / |
| Dosage | 0.25 KWh | 0.002 m^3^ | 0.0042 m^3^ | 0.27 m^3^ | 0.814 Kg | 0.174 Kg | 0.32 Kg | 0.5 Kg | / |
| Cost (＄) | 0.00123 | 2.46 | 0.09 | 0.36 | 1.71 | 1.39 | 26.08 | 1.40 | 32.03 |

Table S4 presents a comparison of different preparation methods. Silicon-carbon materials prepared via the BM method and CVD method are already commercially available, so the cost ranges for these methods were estimated based on market data. However, the HTM method has not yet been commercialized, so no cost estimates are available. Silicon content, waste liquid volume, specific volume, reagent consumption, heating or ball milling time, and water consumption were estimated based on literature [20-22]. The specific capacity indicated in parentheses was estimated in conjunction with other studies. The remaining reference data were estimated by synthesizing three literature sources and published research. In the table, “~” denotes estimated values or ranges, while “/” indicates no relevant data.

**Table S4** Comparison of various preparation methods

| Method | Silicon content (%) | Waste liquid (m^3^) | Specific capacity (mAh/g) | CO_2_ emissions (Kg) | Energy consumption (KWh) | Reagent dosage (m^3^) | Heating or ball milling time (min) | H_2_O (m^3^) | Cost (＄/Kg) |
| --- | --- | --- | --- | --- | --- | --- | --- | --- | --- |
| EICRH | 81.4 | 0.28 | 1800 | -0.513 | 0.25 | 0.0062 | 15 | 0.27 | ~33.63 |
| BM | 14.5 | 0 | 500.1 (500~800) | ~0.05 | 1~8 | 0 | 120 | 0 | 14~22 |
| CVD | 54.4 | ~0.72 | 800 (500~2000) | ~2.25 | 3~10 | ~0.02 | 85 | ~0.7 | 35~50 |
| HTM | ~45.64 | ~1.1 | 701 (600~1800) | ~2 | 1~1.5 | ~0.23 | 180 | ~1 | / |

**References**

[1]Gautam, M., Mishra, G.K., Furquan, M., Bhawana, K., Kumar, D., Mitra, S., Design of Low-Stress robust silicon and Silicon-Carbide anode with high areal capacity and high energy density for Next-Generation Lithium-Ion batteries. *Chemical Engineering Journal* 2023, *472*, 12.

[2]Yang, D.S., Chen, M., Han, R., Luo, Y.T., Li, H., Kang, Z.R., Chen, Y.G., Fu, J., Iqbal, N., Liu, W., Sealing porous carbon via surface-initiated polymerization achieves low-surface-area Si-C microparticles for Li-ion batteries. *Nano Energy* 2024, *127*, 10.

[3]Cheng, Z.L., Lin, H.H., Liu, Y.M., Yan, Q.C., Su, B.L., Zhang, H.J., A Stress-Buffering Hierarchically Porous Silicon/Carbon Composite for High-Energy Lithium-Ion Batteries. *Advanced Functional Materials* 2025, 15.

[4]Lu, J.J., Liu, J.H., Gong, X.Z., Pang, S., Zhou, C.Y., Li, H.X., Qian, G.Y., Wang, Z., Upcycling of photovoltaic silicon waste into ultrahigh areal-loaded silicon nanowire electrodes through electrothermal shock. *Energy Storage Materials* 2022, *46*, 594-604.

[5]Qin, X., Zhao, L., Han, J.W., Xiao, J., Wang, Y.F., Ji, C.Z., Liu, T., Zuo, M.X., Sun, J.S., Kong, D.B., Wu, M.B., Lv, W., Yang, Q.H., Zhi, L.J., Self-Pressure Silicon-Carbon Anodes for Low-External-Pressure Solid-State Li-Ion Batteries. *ACS Nano* 2025, *19* (18), 17760-17773.

[6]Yu, P.L., Li, Z.W., Zhang, D.C., Xiong, Q., Yu, J., Zhi, C.Y., Hierarchical Yolk-Shell Silicon/Carbon Anode Materials Enhanced by Vertical Graphene Sheets for Commercial Lithium-Ion Battery Applications. *Advanced Functional Materials* 2025, *35* (2), 13.

[7]He, J.X., Deng, Y.Z., Han, J.W., Xu, T.Z., Qi, J.S., Li, J.H., Zhang, Y.B., Zhao, Z.Y., Li, Q., Xiao, J., Zhang, J., Kong, D.B., Wei, W., Wu, S.C., Yang, Q.H., Sieving pore design enables stable and fast alloying chemistry of silicon negative electrodes in Li-ion batteries. *Nat. Commun.* 2025, *16* (1), 13.

[8]Kim, M.J., Lee, I., Lee, J.W., Yoon, D., Kim, J.H., Lee, S., Kim, K., Kim, P.J., Choi, J., Kang, Y.C., Jung, D.S., A Novel Structured Si-Based Composite with 2D Structured Graphite for High-Performance Lithium-Ion Batteries. *Small* 2024, *20* (49), 9.

[9]Jin, J.H., Lee, D.G., Kim, J.H., Hong, S., Lim, S., Cho, J.Y., Yoon, J., Kim, J., Park, J.H., Han, J.T., Graphene-Assisted Interfacial Engineering to Develop Binder- and Dispersant-Free Cast Si Alloy/Nanocarbon Anodes for High-Performance Li-Ion Batteries. *Advanced Functional Materials* 2025, 10.

[10]Liu, M.L., Liu, J.W., Jia, Y.Q., Li, C., Zhang, A.W., Hu, R.Z., Liu, J., Wang, C.Y., Ma, L.T., Ouyang, L.Z., Hydrolysis-Engineered Robust Porous Micron Silicon Anode for High-Energy Lithium-Ion Batteries. *Nano-Micro Lett.* 2025, *17* (1), 15.

[11]Cao, L., Xiao, R.S., Wang, J.B., Li, S.Y., Xu, J.J., Huang, T., Recycling Waste Al-Si Alloy for Micrometer-Sized Spongy Si with High Areal/Volumetric Capacity and Stability in Lithium-Ion Batteries. *ACS Sustain. Chem. Eng.* 2022, *10* (25), 8143-8150.

[12]Ju, Z.Y., King, S.T., Xu, X., Zhang, X., Raigama, K.U., Takeuchi, K.J., Marschilok, A.C., Wang, L., Takeuchi, E.S., Yu, G.H., Vertically assembled nanosheet networks for high-density thick battery electrodes. *Proc. Natl. Acad. Sci. U. S. A.* 2022, *119* (40), 9.

[13]Yong, K., Fang, H.Y., Wang, B.Y., Qiu, X.L., Wu, K.P., Wang, Q., Zhang, Y., Wu, H., Synergistic Structural Engineering of Tunnel-Type Polyantimonic Acid Enables Dual-Boosted Volumetric and Areal Lithium Energy Storage. *Advanced Energy Materials* 2022, *12* (26), 15.

[14]Xu, J.H., Yin, Q.Y., Li, X.R., Tan, X.Y., Liu, Q., Lu, X., Cao, B.C., Yuan, X.T., Li, Y.Z., Shen, L., Lu, Y.F., Spheres of Graphene and Carbon Nanotubes Embedding Silicon as Mechanically Resilient Anodes for Lithium-Ion Batteries. *Nano Lett.* 2022, *22* (7), 3054-3061.

[15]Zhao, Z.Y., Han, J.W., Chen, F.Q., Xiao, J., Zhao, Y.F., Zhang, Y.F., Kong, D.B., Weng, Z., Wu, S.C., Yang, Q.H., Liquid Metal Remedies Silicon Microparticulates Toward Highly Stable and Superior Volumetric Lithium Storage. *Advanced Energy Materials* 2022, *12* (7), 8.

[16]Xiao, J., Han, J.W., Kong, D.B., Shi, H.F., Du, X.J., Zhao, Z.Y., Chen, F.Q., Lan, P., Wu, S.C., Zhang, Y.F., Yang, Q.H., "Nano-spring" confined in a shrinkable graphene cage towards self-adaptable high-capacity anodes. *Energy Storage Materials* 2022, *50*, 554-562.

[17]Jin, J.Y., Wang, Z.W., Wang, R., Wang, J.L., Huang, Z.D., Ma, Y.W., Li, H., Wei, S.H., Huang, X., Yan, J.X., Li, S.Z., Huang, W., Achieving High Volumetric Lithium Storage Capacity in Compact Carbon Materials with Controllable Nitrogen Doping. *Advanced Functional Materials* 2019, *29* (12), 9.

[18]Suresh, S., Wu, Z.P., Bartolucci, S.F., Basu, S., Mukherjee, R., Gupta, T., Hundekar, P., Shi, Y.F., Lu, T.M., Koratkar, N., Protecting Silicon Film Anodes in Lithium-Ion Batteries Using an Atomically Thin Graphene Drape. *ACS Nano* 2017, *11* (5), 5051-5061.

[19]Wang, X.H., Sun, L.N., Susantyoko, R.A., Fan, Y., Zhang, Q., Ultrahigh volumetric capacity lithium ion battery anodes with CNT-Si film. *Nano Energy* 2014, *8*, 71-77.

[20]Zhang, H.L., Xu, J.Q., Zhang, J.J., Preparation and electrochemical properties of core-shelled silicon-carbon composites as anode materials for lithium-ion batteries. *J. Appl. Electrochem.* 2019, *49* (11), 1123-1132.

[21]Huang, P., Liu, B., Zhang, J.L., Liu, M.Q., Xie, Z.Y., Silicon/carbon composites based on natural microcrystalline graphite as anode for lithium-ion batteries. *Ionics* 2021, *27* (5), 1957-1966.

[22]Wang, Z.Y., Kong, L.B., Guo, Z.W., Zhang, X.Y., Wang, X.M., Zhang, X., Bamboo-like SiO_x_/C nanotubes with carbon coating as a durable and high-performance anode for lithium-ion battery. *Chemical Engineering Journal* 2022, *428*, 10.
